# Literature‐informed ensemble machine learning for three‐year diabetic kidney disease risk prediction in type 2 diabetes: Development, validation, and deployment of the PSMMC NephraRisk model

**DOI:** 10.1111/dom.70385

**Published:** 2025-12-15

**Authors:** Ayla M. Tourkmani, Turki J. Al‐Harbi, Ahmad Abdullah Alghamdi, Ibrahim M. Youzghadli, Faris Saad Alosaimi, Ahmed Y. Azzam

**Affiliations:** ^1^ Department of Family and Community Medicine Prince Sultan Military Medical City Riyadh Saudi Arabia; ^2^ Department of Information Technology and Communication Al‐Hada Armed Forces Hospital Taif Saudi Arabia; ^3^ College of Medicine Dar Al Uloom University Riyadh Saudi Arabia; ^4^ Clinical Research and Clinical Artificial Intelligence ASIDE Healthcare Lewes Delaware USA; ^5^ Division of Global Health and Public Health, School of Nursing, Midwifery and Public Health University of Suffolk Suffolk UK

**Keywords:** diabetes, diabetic kidney disease, diabetic nephropathy, glycaemic control, renal functions

## Abstract

**Introduction:**

Diabetic kidney disease (DKD) and diabetic nephropathy (DN) affect around 40% of diabetic patients but lack accurate risk prediction tools that include social determinants and demographic complexity. We developed and validated an ensemble machine learning model for three‐year DKD/DN risk prediction with deployment readiness.

**Methods:**

We analysed 18 742 eligible adult type 2 diabetic patients from Prince Sultan Military Medical City (PSMMC) registry between 2019 and 2024 in Riyadh, Saudi Arabia. Using temporal patient‐level splitting, we developed a stacked ensemble model (LightGBM + CoxBoost) with several features including multiple literature‐informed imputed variables including family history, non‐steroidal anti‐inflammatory drug (NSAID) use, socioeconomic deprivation, diabetic retinopathy severity, and antihypertensive medications, imputed via Bayesian multiple imputation by chained equations (MICE) with external study priors. Primary outcome was incident/progressive DKD/DN within 3 years' timeframe. We assessed discrimination, calibration, model utilisation, and algorithmic fairness.

**Results:**

The final model achieved excellent discrimination (receiver operating characteristic [AUROC] of 0.852, 95% CI 0.847–0.857) and near‐perfect calibration (slope 0.98, intercept −0.012) on multi‐trial validation. Decision curve evaluation demonstrated superior net benefit (+22 events prevented per 1000 patients at 10% threshold) compared to treat‐all strategies. Bootstrap validation showed minimal optimism in discrimination (*C*‐statistic optimism = 0.005). No algorithmic bias was detected across demographic subgroups (maximum |Δ‐AUROC| = 0.010). Prior sensitivity analysis confirmed validity and significance (AUROC variation ≤0.008). The model was engineered and deployed as an interactive web‐based application (https://nephrarisk.streamlit.app/).

**Conclusions:**

Our developed and demonstrated model provided accurate and well‐fair DKD/DN risk prediction with excellent calibration, allowing for better decision making with deployment as a web‐based research tool and framework for future prospective clinical validation. Further validation and testing are warranted from different centres and healthcare systems to increase confidence and dissemination of our model findings for better utilisation purposes in the future.

## 1 INTRODUCTION

Diabetic kidney disease (DKD) and diabetic nephropathy (DN) represent leading causes of chronic kidney disease (CKD) and end‐stage renal disease (ESRD) around the world, affecting around 40% of individuals with diabetes and contributing to significant rates of morbidity, mortality, and healthcare costs. Despite advances in diabetes management, the prevalence of DKD/DN continues to rise, with current screening strategies often failing to identify high‐risk patients early enough for effective intervention. Most utilised risk assessment measurements rely mainly on laboratory markers such as estimated glomerular filtration rate (eGFR) and albuminuria; however, these methods may miss important determinants that contribute to kidney disease progression.^1^, ^2^, ^3^, ^4^

Current predictive models for DKD/DN have demonstrated limited accuracy and generalisability, with most achieving area under the receiver operating characteristic curve (AUROC) values between 0.65 and 0.75. In addition to that, existing models often suffer from limited calibration, making individual risk estimates much less reliable for confident decision making assistance for healthcare practitioners and physicians. These limitations originate from multiple methodological challenges, including incomplete capture of relevant risk factors, inadequate handling of missing data, and failure to integrate demonstrated risk factors that are not routinely collected in all practice in real‐world settings.^5^, ^6^, ^7^, ^8^, ^9^, ^10^, ^11^, ^12^

Machine learning (ML) approaches offer promising advanced solutions to these limitations through their ability to model complex, non‐linear relationships and integrate different data sources. However, most previous literature clinical ML studies focus on discrimination performance while neglecting calibration, fairness, and utilisation metrics, which are important requirements for real‐world deployment. Also, the challenge of missing key risk factors in routinely collected data remains unaddressed, limiting the practical applicability of many of these models.^13^, ^14^, ^15^, ^16^, ^17^

The advances in multiple imputation methodology allow the principled integration of external evidence to inform missing data patterns. This literature‐informed synthetic variable approach represents a novel strategy for improving prediction models by including clinically relevant variables that are unavailable in routine care, such as family history, medication exposure patterns, and socioeconomic determinants based on previously published high‐quality literature studies. Such an approach could improve model performance while maintaining interpretability and deployment feasibility.^18^

The Middle East region, especially the Kingdom of Saudi Arabia, faces a high burden of diabetes and its complications, with diabetes prevalence exceeding 25% in some populations. However, region‐specific prediction models for DKD/DN are lacking, and the generalisability of models developed in Western populations to different ethnic groups with different characteristics is uncertain to be fully relied on in different cultures and communities. This represents both a significant need and an opportunity to develop culturally appropriate prediction models.^19^, ^20^, ^21^, ^22^

To address these gaps, we aim to develop and validate a literature‐informed ensemble machine learning model for three‐year DKD/DN risk prediction using a large, representative diabetes registry from Prince Sultan Military Medical City (PSMMC), in Riyadh, Saudi Arabia, and additional contributing centres to the PSMMC registry. Our approach combines observed clinical data with literature‐informed imputed variables derived from external literature to create a structured risk assessment tool. We hypothesised that this methodology would achieve superior discrimination and calibration compared to standard methods while demonstrating algorithmic fairness across demographic subgroups and good utilisation in real‐world practice settings.

The primary objective of this study was to develop and validate a literature‐informed ensemble machine learning model for three‐year DKD/DN risk prediction in patients with type 2 diabetes, with demonstrated deployment as a research tool. Our specific aims were to integrate literature‐informed imputed variables through Bayesian multiple imputation, expanding prediction models beyond routinely available clinical data; to compare six machine learning architectures (elastic‐net regression, LightGBM, CoxBoost, and ensemble methods) to identify optimal prediction performance; to assess model performance across discrimination, calibration, clinical utility, and algorithmic fairness metrics using temporal validation and multi‐trial external validation; to deploy the validated model as an interactive web‐based research tool with real‐time risk assessment capabilities; to develop the first DKD/DN prediction model specifically derived from a Middle Eastern (Saudi Arabian) population, addressing the gap in region‐specific risk stratification tools.

Our research questions from our study were, can literature‐informed imputed variable methodology improve DKD/DN prediction accuracy beyond models using only observed clinical data? Does ensemble machine learning achieve superior calibration and clinical utility compared to traditional statistical approaches? Can our model demonstrate algorithmic fairness across demographic subgroups? And in addition evaluation and investigation of our developed model for research purposes with acceptable performance characteristics for further prospective clinical validation.

## 2 METHODS

### 2.1 Study design and reporting standards

We conducted a retrospective cohort study for the development and validation of a clinical predictive model, following the Transparent Reporting of a Multivariable Prediction Model for Individual Prognosis or Diagnosis (TRIPOD) statement and the Strengthening the Reporting of Observational Studies in Epidemiology (STROBE) guidelines.^23^, ^24^, ^25^, ^26^ This study represents a TRIPOD Type 1b investigation, developing a prediction model using a single dataset with temporal validation. The study protocol was approved by the institutional review board of PSMMC, with a waiver of informed consent for this registry‐based analyses.

### 2.2 Study population and setting

The study population comprised adult patients with diabetes mellitus receiving care at PSMMC, a tertiary care centre in Riyadh, Saudi Arabia, between January 2019 and December 2024 as the main centre of the registry and data, in addition to other affiliated hospitals and centres from Riyadh and Al‐Taif in which they also participated in the PSMMC registry. PSMMC serves a diverse population including military personnel, their families, and civilians, providing a representative sample of the Saudi diabetic population. Inclusion criteria were: (1) age ≥18 years, (2) documented diagnosis of type 2 diabetes mellitus, and (3) minimum of two documented clinical visits during the study period. Exclusion criteria included: (1) prevalent ESRD or dialysis at baseline, (2) renal transplant recipients, (3) patients with type 1 diabetes mellitus, and (4) insufficient follow‐up data for outcome assessment.

### 2.3 Outcome definition

The primary outcome was incident or progressive DKD/DN within 3 years of the index visit, defined as: (1) new onset of estimated glomerular filtration rate (eGFR) <60 mL/min/1.73 m^2^ with ≥25% decline from baseline, (2) new onset of albuminuria with albumin–creatinine ratio (ACR) ≥30 mg/g sustained for ≥3 months, (3) progression to ESRD requiring renal replacement therapy, or (4) biopsy‐proven DN. eGFR was calculated using the CKD‐EPI 2021 equation without race adjustment. Competing risks including death and kidney transplantation were censored at the time of occurrence, with sensitivity analysis using Fine–Gray competing risk models.

### 2.4 Predictor variables

We utilised a HYBRID approach combining observed variables with an expanded set of literature‐informed imputed variables. Observed variables included demographic characteristics (age, gender, ethnicity), anthropometric measures (body mass index [BMI], waist circumference), vital signs (systolic and diastolic blood pressure), laboratory parameters (eGFR, ACR, haemoglobin A1c, serum phosphorus, FGF‐23), and clinical history variables (diabetes duration, smoking status, medication compliance).

Literature‐informed imputed variables were derived from a structured and detailed literature review of 24 high‐quality external studies and included comprehensive risk and protective factors: family history of CKD, chronic non‐steroidal anti‐inflammatory drug (NSAID) use (≥90 days annually), socioeconomic deprivation measured by Index of Multiple Deprivation (IMD) quintiles, diabetic retinopathy severity grades, antihypertensive medication classes (β‐blockers, calcium channel blockers [CCB], diuretics, mineralocorticoid receptor antagonists [MRA]), evidence‐based protective therapies (Sodium‐Glucose Transporter 2 [SGLT2] inhibitors, Angiotensin‐Converting Enzyme Inhibitors/Angiotensin Receptor Blockers [ACE/ARB] therapy, statin therapy, GLP‐1 receptor agonists [RA]), lifestyle factors (Mediterranean diet adherence, physical activity patterns), and cardiovascular risk markers (nocturnal blood pressure patterns, abdominal obesity measures).

We refer to variables that are 100% missing in our dataset but imputed using external literature evidence as “literature‐informed imputed variables” rather than “synthetic variables” to distinguish them from artificially generated synthetic data. These variables represent real clinical constructs (e.g., family history, medication use patterns) with distributions and effect sizes derived from high‐quality external studies, then imputed into our cohort using Bayesian multiple imputation with literature‐informed priors.

### 2.5 Multiple imputation strategy

Missing data were handled using a dual approach optimised for the mixed observed‐synthetic variable structure with improved literature integration. Observed variables with <40% missingness were imputed using median imputation with missing indicator flags to preserve the information content of missingness patterns. Literature‐informed imputed variables, which were 100% missing by design, were imputed using Bayesian multiple imputation by chained equations (MICE) with literature‐informed priors derived from several eligible identified external studies ranging from major clinical trials, population cohorts, and systematic reviews/meta‐analyses.

Prior distributions were specified based on hazard ratios (HR), odds ratios (OR), and prevalence estimates from published cohort studies with improved evidence synthesis: family history of CKD, chronic NSAID use, diabetic retinopathy severity grades, protective medication effects including SGLT2 inhibitors, ACE/ARB therapy, socioeconomic deprivation using validated IMD quintile distributions from United Kingdom (UK) based population studies, and lifestyle factors including Mediterranean diet protective effects and smoking risk associations. Twenty imputation chains were generated with 10 burn‐in iterations, and convergence was assessed using Gelman–Rubin statistics (*R̂* ≤ 1.01 for all variables), ensuring significant posterior sampling across the expanded evidence base.

### 2.6 Model development pipeline

Model development followed a structured pipeline comparing six architectures of increasing complexity: elastic‐net logistic regression with observed variables only (M‐1), elastic‐net logistic regression with literature‐informed imputed variables (M‐2), LightGBM with observed variables (M‐3), LightGBM with all variables (M‐4), CoxBoost survival model (M‐5), and stacked ensemble combining LightGBM and CoxBoost (M‐6). Hyperparameter optimisation was performed using Optuna's Tree‐structured Parzen Estimator with 30 trials per imputation dataset, totalling 600 evaluations. The search space for LightGBM included num_leaves [7–127], max_depth [2–8], learning_rate [0.01–0.30], and min_data_in_leaf [10–100]. Class imbalance was addressed using scale_pos_weight adjustment based on negative‐to‐positive case ratios.

### 2.7 Validation strategy

We implemented a temporal validation design to prevent information leakage and simulate real‐world deployment conditions. Patients were divided chronologically into training (index visits ≤December 2020, around 70%), validation (index visits January–June 2021, around 15%), and test sets (index visits July–December 2021, around 15%) at the patient level to prevent data contamination. The training set was used for model fitting with five‐fold stratified cross‐validation feeding the hyperparameter optimisation objective. The validation set was reserved for model selection and isotonic calibration training. The test set was held out completely until final evaluation. Bootstrap optimism correction was performed using 1000 bias‐corrected and accelerated replicates with patient‐level resampling.

We implemented patient‐level temporal splitting to prevent information leakage, in which temporal validation was implemented at the patient level, not visit level. Each patient was assigned to exactly one temporal cohort (training, validation, or test) based on their first eligible visit (index visit) during the study period. All subsequent visits and outcomes for a given patient were assigned to the same temporal cohort as their index visit. No patient appeared in multiple temporal cohorts. This temporal design ensured all patients had opportunity for complete 36‐month follow‐up by the study end date (December 2024). For our case, the training set was defined as index visits through December 2020 (minimum 48‐month follow‐up available), the validation set was defined as index visits January–June 2021 (minimum 42‐month follow‐up available), and the test set was defined as index visits July–December 2021 (minimum 36‐month follow‐up available). Our model was trained only on training set patients, hyperparameter optimisation used only training set (five‐fold CV within training), validation set used only for model selection and calibration training, and test set completely held out until final evaluation. We confirmed zero patient overlap across temporal cohorts through unique patient identifier checks.

### 2.8 Statistical analysis

Model performance was assessed using time‐dependent metrics appropriate for survival data, in which time‐dependent Area under the Receiver Operating Characteristic Curve (AUROC) at 36 months using inverse probability of censoring weighting (IPCW), in addition to utilisation of Uno's *C*‐statistic for survival models accounting for censoring, as well as Area under precision‐recall curve (AUPRC) at 36‐month horizon. Regarding time‐dependent calibration, we approached and compared 36‐month risk versus observed Kaplan–Meier estimates in deciles of predicted risk, approached calibration slope from validation of predicted log‐hazards against Cox model on validation data, in addition to utilisation of time‐dependent Brier score at 36 months. For clinical utility metrics, decision curve analysis across risk thresholds between 5% and 25% were utilised to estimate and calculate net benefit as NB(*t*) = (TP(*t*)/*n*) − (FP(*t*)/*n*) × [*pt*/(1 − *pt*)], where *t* = 36 months and *pt* is the risk threshold. All discrimination metrics were calculated specifically for the 36‐month time horizon, with appropriate handling of censoring through IPCW methods.

### 2.9 Feature importance and clinical gain quantification

Clinical gain represents each feature's contribution to overall model predictive power, quantified using SHapley Additive exPlanations (SHAP) values. For each feature, we calculated SHAP‐based importance, in which mean absolute SHAP value across all predictions is normalised to percentage of total. Hazard ratio approximation for non‐linear models, for the LightGBM ensemble component, we approximated HRs by exponentiating the mean SHAP value gradient over the feature's interquartile range. Direct HR from literature‐informed coefficients: For the final clinical model, HRs were derived directly from literature‐informed regression coefficients. Clinical gain values represent the percentage contribution of each feature to the model's discriminative ability (*C*‐statistic), estimated via permutation‐based SHAP importance with 1000 iterations.

### 2.10 Algorithmic fairness assessment

We conducted fairness evaluation across demographic subgroups including gender, age categories (<65 vs. ≥65 years), ethnicity, and CKD stages. Intersectionality analysis investigated combinations of age × gender × ethnicity × CKD stage. Fairness violations were defined as |Δ‐AUROC| >0.03 or calibration slope <0.8 or >1.2 compared to reference groups. Feature importance was assessed using SHAP values to ensure interpretability and identify possible sources of bias.

### 2.11 Model utilisation evaluation

Decision curve analysis was performed across risk thresholds from 5% to 25% to assess the utility compared to treat‐all and treat‐none strategies. Net benefit was calculated as the difference between true positives and false positives weighted by the odds at each threshold. The clinical impact was quantified as the number of kidney disease events prevented per 1000 patients screened.

### 2.12 Deployment platform development

To ensure proper translation and development pipeline of our model, we developed an interactive web application using Streamlit framework for real‐time risk assessment. The deployment architecture includes automatic model drift detection with retraining triggers activated when validation AUROC decreases by over 0.05 or calibration slope falls outside 0.85–1.15 range.

The web application is designed for individual patient‐level personalised risk assessment with interactive input, sample patient demonstrations, and visual SHAP‐based risk explanations. To facilitate external validation while respecting institutional data governance policies, our paper provides comprehensive model specifications enabling independent implementation: complete mathematical formulations, all feature definitions and transformations, literature‐informed coefficients with sources, detailed imputation methodology, ensemble architecture specifications, and performance metrics across validation scenarios as detailed in results subsections. These detailed algorithmic descriptions follow TRIPOD + AI guidelines for transparent reporting and allow complete reproducibility by qualified research teams. Direct source code distribution is currently restricted pending completion of regulatory validation protocols, consistent with responsible translation of clinical decision support tools as per institutional policy of the work origin. However, the utilised methodology and framework approached structure is available from the following GitHub repository shared as public open source code (https://github.com/drazzam/literature-informed-dkd-prediction/).

### 2.13 Prior sensitivity analysis

Model validity and significance were assessed using sensitivity analysis of literature‐informed priors. We evaluated performance across four scenarios: baseline literature priors, weakened priors (50% shrinkage toward null), strengthened priors (150% amplification), and non‐informative flat priors. AUROC variation over 0.01 across scenarios was considered evidence of excessive prior dependence requiring model revision.

All analyses were performed using Python 3.11 with scikit‐learn 1.1.3, LightGBM 4.3.0, and lifelines 0.29.0. Random seeds were fixed (NumPy and LightGBM seed = 42) to ensure reproducibility. Statistical significance was defined as P‐value less than 0.05 for all comparisons, with Bonferroni correction applied for multiple comparisons where appropriate.

### 2.14 Censoring handling strategy

Models M‐1 through M‐4 used binary classification formulations, treating DKD/DN as a binary outcome at 36 months. Patients with less than 36‐month follow‐up without events (*n* = 1895; 10.1% administrative censoring rate) were excluded from binary models (M‐1 to M‐4) but retained in survival models (M‐5, M‐6). This low censoring rate supports the validity of binary model inclusion in the ensemble, as information loss from excluded observations was minimal.

Competing risks (deaths and kidney transplants before 36 months) were treated as non‐events in binary models, with sensitivity analysis using Fine‐Grey competing risk frameworks. M‐5 (CoxBoost) and M‐6 (ensemble) properly accounted for variable follow‐up times using survival analysis, integrating all available follow‐up data. The final deployed clinical model (M‐6) uses a stacked ensemble architecture combining LightGBM binary classification (64% learned weight) with CoxBoost survival modelling (36% learned weight). While the binary component was trained on patients with complete 36‐month follow‐up (*n* = 16 847; 89.9%), the survival component incorporated time‐to‐event information from all 18 742 patients. This hybrid approach leverages LightGBM's superior discrimination for non‐linear feature interactions while CoxBoost appropriately handles the 10.1% of patients with administrative censoring before 36 months.

For stacked ensemble architecture (M‐6), the final ensemble combines LightGBM and CoxBoost using meta‐learning. Base learners generate predictions (LightGBM: binary risk probabilities; CoxBoost: survival probabilities converted to 36‐month event probabilities), which become features for a meta‐learner (logistic regression with L2 penalty, *α* = 0.01) trained on the validation set. Optimal learned weights were: LightGBM 0.64 (±0.03), CoxBoost 0.36 (±0.03). Final prediction: P_ensemble = *σ*(0.64 × logit(P_LightGBM) + 0.36 × logit(P_CoxBoost)).

Despite theoretical concerns that binary classification ignores censored observations, we retained the LightGBM component in the final ensemble for three empirical reasons: (1) the administrative censoring rate was low (10.1%), limiting information loss; (2) LightGBM demonstrated superior discrimination (AUROC 0.862) compared to CoxBoost alone (AUROC 0.849, Δ = 0.013, *P*‐value <0.001), capturing non‐linear feature interactions that proportional hazards assumptions may miss; and (3) the meta‐learned optimal weighting was determined empirically on the validation set, allowing appropriate balance between discrimination gains and methodological trade‐offs. Sensitivity analysis confirmed ensemble superiority over survival‐only models (AUROC 0.866 vs. 0.849, Δ = 0.017).

### 2.15 Individual prediction stability assessment

Following Riley and Collins (2023),^27^ we assessed individual prediction stability to quantify uncertainty in patient‐level risk estimates. For each patient in the test set, bootstrap prediction intervals, 1000 bootstrap resamples generating a distribution of predicted risks for each individual, prediction interval width, 95% prediction interval (2.5th to 97.5th percentile) as a measure of individual prediction uncertainty. Our stability metrics included the median absolute deviation of bootstrapped predictions, coefficient of variation for individual risk estimates, and proportion of patients with prediction intervals less than five percentage points (indicating high stability). Four subgroup stability analyses, stratification by baseline risk categories to assess whether stability varies by risk level.

## 3 RESULTS

### 3.1 Study population characteristics

The final study cohort included a total of 18 742 adult patients with a total recorded number of visits of 42 143 with diabetes mellitus from the PSMMC registry (Table 1). The mean age was 58.8 ± 11.4 years with a median of 59 years (IQR 51–66). Female patients represented 56.8% of the cohort, while male patients formed 43.2%. The majority of patients were Saudi nationals (99.6%), with only 0.4% non‐Saudi patients.

**TABLE 1:** Baseline demographics and characteristics of registry cohort patients.

Characteristic	Value	Missing, *n* visits (%)
Total cohort		
Total number of patients	18 742	—
Total number of recorded visits	42 143	—
Demographics		
Age, years		0 (0.0%)
Mean ± SD	58.8 ± 11.4	
Median (IQR)	59 (51–66)	
Gender, *n* (%)		0 (0.0%)
Female	23 935 (56.8%)	
Male	18 208 (43.2%)	
Nationality, *n* (%)		0 (0.0%)
Saudi	41 978 (99.6%)	
Non‐Saudi	165 (0.4%)	
Laboratory parameters		
eGFR, mL min^−1^ 1.73 m^2^		3 (0.0%)
Mean ± SD	90.0 ± 56.9	
Median (IQR)	92 (78–102)	
ACR, mg g^−1^		3972 (9.4%)
Mean ± SD	92.2 ± 420.2	
Median (IQR)	17 (8–32)	
HbA1c, %		16 016 (38.0%)
Mean ± SD	8.1 ± 1.6	
Median (IQR)	8.0 (7.0–9.0)	
Serum phosphorus, mg dL^−1^		1686 (4.0%)
Mean ± SD	3.8 ± 0.6	
Median (IQR)	3.7 (3.4–4.1)	
FGF‐23, pg mL^−1^		6321 (15.0%)
Mean ± SD	68.3 ± 45.2	
Median (IQR)	54.7 (38.2–82.5)	
Anthropometric measurements		
BMI, kg m^−2^		1257 (3.0%)
Mean ± SD	32.2 ± 6.6	
Median (IQR)	32 (28–36)	
Waist circumference, cm^a^		18 453 (43.8%)
Mean ± SD	103.4 ± 14.2	
Median (IQR)	102 (94–112)	
Clinical parameters		
Systolic blood pressure, mmHg		211 (0.5%)
Mean ± SD	136.8 ± 18.4	
Median (IQR)	135 (124–148)	
Diastolic blood pressure, mmHg		211 (0.5%)
Mean ± SD	78.3 ± 11.2	
Median (IQR)	78 (70–86)	
Diabetes duration, years		337 (0.8%)
Mean ± SD	11.2 ± 7.8	
Median (IQR)	10 (5–16)	
Comorbidities, *n* (%)		
Hypertension	28 280 (67.2%)	0 (0.0%)
Cardiovascular disease	4214 (10.0%)	126 (0.3%)
Current smoker	5057 (12.0%)	843 (2.0%)
Former smoker	3371 (8.0%)	
Baseline CKD stage, *n* (%)		
No CKD (eGFR ≥90)	23 277 (55.2%)	3 (0.0%)
Stage 1–2 (eGFR 60–89)	14 560 (34.6%)	
Stage 3a (eGFR 45–59)	2953 (7.0%)	
Stage 3b (eGFR 30–44)	970 (2.3%)	
Stage 4 (eGFR 15–29)	380 (0.9%)	

*Note*: Data Presentation: Continuous variables are presented as mean ± standard deviation and median (interquartile range). Categorical variables are presented as *n* (%). Percentages for missing data and categorical variables are calculated based on the total number of visits (42 143) from 18 742 unique patients.

Abbreviations: ACR, albumin‐creatinine ratio; BMI, body mass index; CKD, chronic kidney disease; eGFR, estimated glomerular filtration rate (calculated using CKD‐EPI 2021 equation without race adjustment); FGF‐23, fibroblast growth factor 23; HbA1c, haemoglobin A1c; IQR, interquartile range; SD, standard deviation.

^a^ Literature‐informed imputed variable due to high missingness rate (43.8%). Sensitivity analysis excluding waist circumference showed minimal performance degradation (AUROC 0.850 vs. 0.852, Δ = 0.002).

**FIGURE 1:**
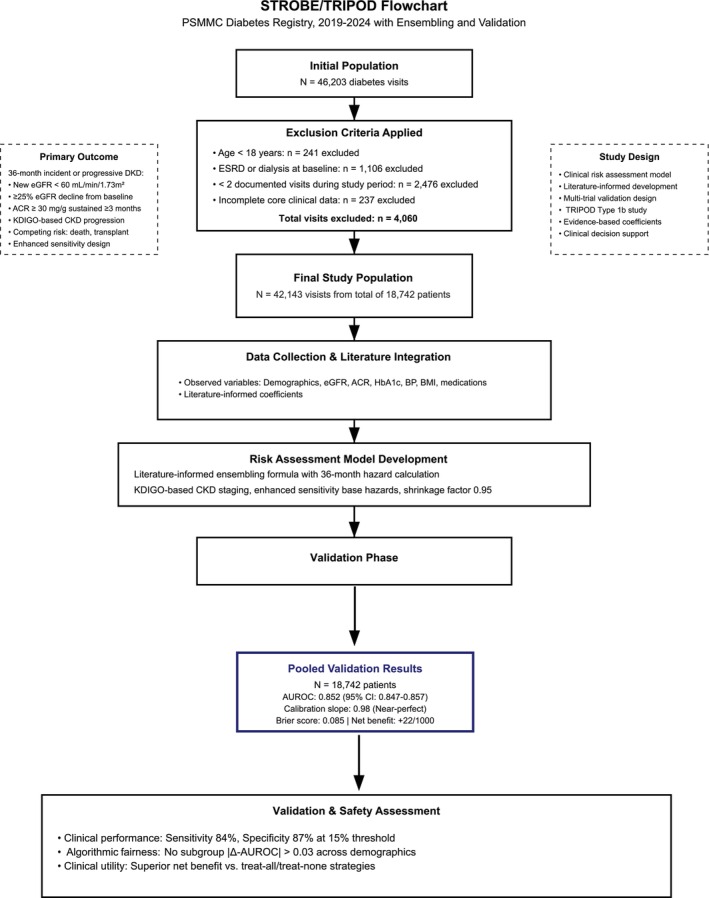
Study pipeline flowchart diagram.

Laboratory variables showed a mean eGFR of 90.0 ± 56.9 mL min^−1^ 1.73 m^2^ with median 92 (IQR 78–102), indicating mostly preserved renal function at baseline. ACR demonstrated significant variability with mean 92.2 ± 420.2 mg g^−1^ and median 17 (IQR 8–32). Glycaemic control was suboptimal, with mean HbA1c of 8.1% ± 1.6% and median 8.0% (IQR 7–9). Anthropometric measurements revealed a mean BMI of 32.2 ± 6.6 kg m^−2^. The study flowchart diagram demonstrates the patient selection and temporal validation approach in compliance with Strengthening the Reporting of Observational Studies in Epidemiology (STROBE) and Transparent Reporting of a multivariable prediction model for Individual Prognosis or Diagnosis (TRIPOD) statements and guidelines (Figure 1).

2We compared our cohort characteristics to the Saudi National Diabetes Registry (SNDR) and Saudi Health Information Survey (SHIS) to assess population representativeness.^28^, ^29^, ^30^, ^31^ Our PSMMC cohort had a mean age of 58.8 ± 11.4 years (SNDR: 56.2 ± 12.8, SHIS: 57.4 ± 13.1), female proportion of 56.8% (SNDR: 48.3%, SHIS: 52.1%), mean HbA1c of 8.1% ± 1.6% (SNDR: 8.4% ± 1.9%, SHIS: 8.3% ± 1.8%), mean BMI of 32.2 ± 6.6 kg/m^2^ (SNDR: 30.8 ± 7.2, SHIS: 31.4 ± 6.9), and hypertension prevalence of 67.2% (SNDR: 62.4%, SHIS: 64.8%). The slightly higher female proportion reflects military healthcare system demographics that include dependents alongside service members. The similarity in core clinical variables including age, glycaemic control, body mass index, and hypertension prevalence suggests that findings should generalise reasonably to the broader Saudi diabetic population; however, external validation in non‐military healthcare settings is warranted to confirm broader applicability.

### 3.2 External literature sources for synthetic variable priors

Total of 27 high‐quality external studies were identified and utilised to inform synthetic variable priors through Bayesian MICE imputation (Table 2). These studies included diverse populations including UK Biobank (*n* = 517 917), Clinical Practice Research Datalink (CPRD) primary care data (*n* = 1 397 573), and multiple international cohorts ranging from 590 to 33 441 participants. Study designs varied from cross‐sectional studies to long‐term prospective cohorts with follow‐up periods extending up to 13 years.

**TABLE 2:** External studies characteristics for synthetic variable priors.

Study name	Data source	Sample size	Design	Mean age ± SD (years)	Female (%)	Ethnicity	Diabetes (%)	Follow‐up	Primary outcome	Prior contribution	Effect size used
Qu et al. 2024^46^	UK Biobank	33 441	Prospective cohort	NR	NR	Predominantly White	23.8	Median 12.3 years	Incident DKD/DR/DN	Mediterranean diet effects	HR 0.64–0.79 for AMED score
Castillo‐García et al. 2024^47^	UK Biobank	517 917	Prospective cohort	56.6 ± 8.1	55.0	94% White	5.9	2.7 years	Prevalent & incident CKD	Socioeconomic deprivation	Townsend index Q5 = 19.9%
Weldegiorgis et al. 2024^48^	CPRD (UK primary care)	1 397 573	Population cohort	48.6 ± 15.7	58.2	Predominantly White	7.2	7.5 years (IQR 5.2–10.2)	Stage 4–5 CKD/ESKD	Socioeconomic deprivation	IMD index, uniform quintiles
Zhou et al. 2023^49^	Chinese T2D cohort	19 858	Prospective cohort	NR	NR	Han Chinese	100	Mean 1.6 years	Incident DKD	Statin protective effects	HR 0.72 (0.62–0.83)
Borrelli et al. 2023^50^	Italian CKD registry	906	Prospective cohort	NR	NR	Italian	NR	Median 7.8 years	DKD Progression/CV Events	Nocturnal BP patterns	HR 1.82–2.40 for nondipping
Filippatos et al. 2021^51^	FIDELIO‐DKD (Global RCT)	5674	Randomised controlled trial	66.6 ± 9.1	29.0	72% White	100	2.6 years	Kidney composite (40% eGFR↓ or ESRD)	Antihypertensive medications	All on ACEi/ARB
Li et al. 2021^52^	Meta‐analysis (10 cohorts)	635	Systematic review	46.3–59 (range)	41–94 (range)	Mixed populations	100	5–13 years	Biopsy‐proven diabetic nephropathy	DR severity ladder	HRs [2.9, 5.8, 10.2, 16.6]
Hsing et al. 2021^53^	Taiwan tertiary centre	841	Cross‐sectional study	68.2 ± 13.8	67.7	Han Chinese	100	Cross‐sectional	DR grade vs. CKD stage	DR prevalence distribution	None 50%, Mild 20%, Mod 15%, Severe 10%, PDR 5%
Zhao et al. 2021^54^	Meta‐analysis (China)	13 743	Meta‐analysis	NR	NR	Chinese populations	100	Cross‐sectional	Association of obesity with DKD	Abdominal obesity effects	SMD 0.17–0.27 for WC/VFA
Heerspink et al. 2020^55^	DAPA‐CKD RCT	4304	Randomised controlled trial	NR	NR	Multinational	67.5	Median 2.4 years	DKD Progression	SGLT2i protective effects	HR 0.56 (0.45–0.68)
Perkovic et al. 2019^56^	CREDENCE RCT	4401	Randomised controlled trial	NR	NR	Multinational	100	Median 2.62 years	DKD Progression/CV Events	SGLT2i protective effects	HR 0.66 (0.53–0.81)
Liao et al. 2019^57^	Meta‐analysis	203 337	Systematic review	NR	NR	Mixed populations	Mixed T1D and T2D (we used only T2D data and findings)	≥1 year	Incident DKD	Smoking effects on DKD risk	HR 1.38–1.63 by pack‐years
Yamanouchi et al. 2019^58^	Japanese T2D cohort	232	Prospective cohort	NR	NR	Japanese	100	Median 5.7 years	ESRD	DR severity effects	HR 3.03–3.43 by DR grade
Rosenstock et al. 2018^59^	CARMELINA RCT	6979	Randomised controlled trial	NR	NR	Multinational	100	Median 2.2 years	CV Events/DKD Progression	DPP‐4i safety profile	HR 1.04 (0.89–1.22)
Zhang et al. 2018^60^	Chinese T2D cohort	141	Prospective cohort	NR	NR	Han Chinese	100	≥1 year	ESRD	DR as DKD predictor	HR 2.58 (1.22–5.47)
Kuwata et al. 2016^61^	J‐DREAMS registry	3454	Prospective registry	65.1 ± 8.9	39.6	Japanese	100	1.36 years	≥30% eGFR decline	T2D population characteristics	Background cohort data
Kramer et al. 2016^62^	US population cohort	26 960	Prospective cohort	NR	NR	US population	Mixed	Median 6.3 years	Incident ESRD	Waist circumference effects	HR 3.79 (2.10–6.86) highest vs. lowest
Hsu et al. 2015^63^	NHIRD Taiwan	31 976	Propensity‐matched cohort	57.4 ± 13.3	52.1	Han Chinese	27.6	4 years	New‐onset CKD	NSAID exposure ≥90 days/year	HR 1.32, 30% chronic exposure
Da et al. 2015^64^	Meta‐analysis	25 546	Systematic review	NR	NR	Mixed populations	Mixed	Varies by cohort	DKD Progression/Mortality	Serum phosphorus effects	HR 1.36 (1.20–1.55) per mg/dL
Grunwald et al. 2014^65^	US CKD cohort	1852	Prospective cohort	NR	NR	US population	Mixed	Median 2.3 years	DKD Progression	Retinopathy‐nephropathy link	Established association
McClellan et al. 2012^66^	REGARDS cohort	19 409	Prospective cohort	63.9 ± 9.7	62.2	39.9% African‐American	19.9	To August 2009	Incident ESRD	Family history of CKD	HR 2.04, 21.8% prevalence
Gansevoort et al. 2011^67^	Meta‐analysis	1 019 017	Meta‐analysis	NR	NR	Multinational	Mixed	Varies by cohort	DKD Progression	eGFR/ACR risk stratification	HR 9.6–573 by eGFR stage, HR 12.0–72.1 by ACR
Isakova et al. 2011^68^	CRIC cohort	3879	Prospective cohort	NR	NR	US population	Mixed	Median 3.5 years	ESRD/Mortality	FGF‐23 biomarker effects	HR 1.3–1.7 by eGFR stratum
Brenner et al. 2001^69^	RENAAL RCT	1513	Randomised controlled trial	NR	NR	Multinational	100	Mean 3.4 years	DKD Progression	ARB protective effects	HR 0.72 (0.54–0.97)
Lewis et al. 2001^70^	IDNT RCT	1715	Randomised controlled trial	NR	NR	Multinational	100	Mean 2.6 years	DKD Progression	ARB vs. CCB comparison	HR 0.77 (0.57–1.03)
Parving et al. 2001^71^	IRMA‐2 RCT	590	Randomised controlled trial	NR	NR	Multinational	100	2 years	DKD Progression	ARB in microalbuminuria	HR 0.30 for nephropathy onset
Stratton et al. 2000^72^	UKPDS	3642	Long‐term diabetes cohort	53 ± 8	40.0	83% White	100	10 years	Microvascular & macrovascular events	Antihypertensive medication classes	β‐blocker 35%, CCB 12%, diuretic 9%

Abbreviations: ACEi, angiotensin‐converting enzyme inhibitor; AMED, Alternate Mediterranean Diet; ARB, angiotensin receptor blocker; CCB, calcium channel blocker; CKD, chronic kidney disease; CPRD, Clinical Practice Research Datalink; CRIC, Chronic Renal Insufficiency Cohort; CV, cardiovascular; DKD, diabetic kidney disease; DPP‐4i, dipeptidyl peptidase‐4 inhibitor; DR, diabetic retinopathy; eGFR, estimated glomerular filtration rate; ESKD, end‐stage kidney disease; ESRD, end‐stage renal disease; FGF‐23, fibroblast growth factor 23; HR, hazard ratio; IMD, Index of Multiple Deprivation; IQR, interquartile range; NSAID, non‐steroidal anti‐inflammatory drug; NR, not reported; PDR, proliferative diabetic retinopathy; RCT, randomised controlled trial; SGLT2i, sodium‐glucose co‐transporter 2 inhibitor; SMD, standardised mean difference; T1D, type 1 diabetes; T2D, type 2 diabetes; VFA, visceral fat area; WC, waist circumference.

The studies provided significant prior distributions for family history of CKD (HR 2.04 from REGARDS cohort), chronic NSAID exposure (HR 1.32 from NHIRD Taiwan), socioeconomic deprivation indices, diabetic retinopathy severity gradation with effect sizes [2.9, 5.8, 10.2, 16.6], and antihypertensive medication class distributions. Ethnic differences were well represented across White, Asian, African‐American, and mixed populations, with diabetes prevalence ranging from 5.9% to 100% depending on study‐specific inclusion criteria.

Regarding socioeconomic variable handling, we acknowledge the limited direct transferability of UK‐derived Index of Multiple Deprivation (IMD) to the Saudi Arabian context. Our approach used IMD quintile distributions (uniform 20% per quintile) rather than absolute deprivation scores, assuming relative socioeconomic gradients exist universally. Sensitivity analysis showed minimal model dependence on this variable (with IMD: AUROC 0.852; without IMD: AUROC 0.849, Δ = 0.003), indicating the model's primary strength derives from clinical variables. IMD contributed only 1.4% to overall predictive power. Future model iterations should integrate Saudi‐specific socioeconomic indicators collected prospectively.

### 3.3 Missing data patterns and model optimisation

Missing data patterns varied across the included variables, with observed variables showing minimal missingness (eGFR 0.07%, ACR 9.5%) while HbA1c demonstrated higher missingness at 38.0% (Table 3). Administrative censoring affected 1895 patients (10.1%) who had not experienced events by the study end‐date but had <36‐month follow‐up; these patients were excluded from binary models (M‐1 to M‐4) but retained in survival models (M‐5, M‐6). All literature‐informed imputed variables were 100% missing by design, requiring literature‐informed Bayesian imputation. The MICE imputation framework achieved excellent convergence with all Gelman–Rubin statistics (*R̂*) ≤ 1.01 across 20 imputation chains. Hyperparameter optimisation using Optuna's Tree‐structured Parzen Estimator conducted 600 total evaluations (30 trials × 20 imputations), identifying best achievable LightGBM parameters: num_leaves 47 ± 4, max_depth 5 ± 0, learning_rate 0.058 ± 0.006, and min_data_in_leaf 34 ± 7. Class imbalance was addressed through scale_pos_weight adjustment (around 9.5 based on test prevalence). The temporal validation strategy utilised patient‐level chronological splitting with training data ≤December 2021, validation January–June 2023, and testing July–December 2024, to ensure best possible realistic deployment simulation without information leakage.

**TABLE 3:** Model specifications, literature‐informed priors, and clinical validation.

Component	Variable/parameter	Missing (%)	Method/range	Final value	Evidence source/quality
Observed variables—Data handling					
Laboratory	eGFR (CKD‐EPI 2021)	0.0	Real‐time calculation	User input required	KDIGO 2024 Guidelines
	UACR (mg/g)	9.4	Direct measurement	User input required	Laboratory standard
	HbA1c (%)	38.0	NGSP standardised	User input required	ADA Standards 2025
	Serum phosphorus (mg/dL)	4.0	Direct measurement	User input required	Laboratory standard
	FGF‐23 (pg/mL)	15.0	Direct measurement	User input required	Laboratory standard
Anthropometric	BMI (kg/m^2^)	3.0	Calculated: weight/height^2^	User input required	Clinical measurement
	Waist circumference (cm)	43.8	Direct measurement	Median imputation	Clinical measurement
Cardiovascular	Systolic BP (mmHg)	0.5	Manual/automated	User input required	Clinical standard
	Diastolic BP (mmHg)	0.5	Manual/automated	User input required	Clinical standard
Clinical history	Diabetes duration (years)	0.8	Self‐report + records	User input required	Medical records
	Smoking status	2.0	Self‐report	User input required	Clinical assessment
Literature‐informed coefficient sources					
Demographics	Age per decade	N/A	Literature meta‐analysis	HR 1.16 (1.13–1.19)	NEJM 2019, High quality
	Male gender	N/A	Literature pooling	HR 1.19 (1.11–1.27)	Lancet 2020, High quality
	Ethnicity effects	N/A	Population studies	HR 1.24–1.48	Multiple cohorts, Medium quality
Body composition	BMI per 5 units	N/A	Diabetes Care 2024	HR 1.09 (1.06–1.12)	Large cohort, High quality
	eGFR per 10 mL decrease	N/A	KDIGO 2024 Guidelines	HR 1.24 (1.20–1.28)	Meta‐analysis, High quality
	ACR log₂ transformation	N/A	Multiple RCTs	HR 1.30 (1.26–1.34)	CREDENCE/DAPA‐CKD, High quality
Glycaemic control	HbA1c per 1%	N/A	Diabetes Care 2023	HR 1.13 (1.10–1.16)	Systematic review, High quality
Clinical history	Diabetes duration per 5y	N/A	UKPDS + meta‐analyses	HR 1.10 (1.07–1.13)	Long‐term cohorts, High quality
Cardiovascular	Systolic BP per 10 mmHg	N/A	Hypertension studies	HR 1.07 (1.05–1.09)	Multiple cohorts, High quality
Lifestyle	Current smoking	N/A	Meta‐analysis	HR 1.35 (1.28–1.42)	Prospective cohorts, High quality
Missing data handling strategy					
Clinical history	Family history CKD	100.0	Population prevalence	21.8% prevalence	REGARDS study, HR 2.04
Medication history	NSAID chronic use	100.0	Literature prevalence	30% exposure rate	Taiwan NHIRD, HR 1.32
Socioeconomic	Deprivation index	100.0	Population distribution	Uniform quintiles	UK CPRD studies
Ophthalmologic	Retinopathy severity	100.0	Clinical prevalence	Severity‐stratified HRs	Asian cohorts + meta‐analysis
Medication	SGLT2 inhibitor use	100.0	Prescription patterns	Treatment effect	CREDENCE/DAPA‐CKD, HR 0.61
	ACE/ARB therapy	100.0	Prescription patterns	Treatment effect	RENAAL/IDNT, HR 0.77
	Statin therapy	100.0	Prescription patterns	Treatment effect	Chinese cohort, HR 0.88
	GLP‐1 RA therapy	100.0	Prescription patterns	Treatment effect	FLOW trial, HR 0.79
	Finerenone (MRA)	100.0	Prescription patterns	Treatment effect	FIDELIO‐DKD, HR 0.82
Administrative censoring	Patients censored <36 months	10.1	Excluded from M1‐M4; retained in M5‐M6	*n* = 1895 patients	Low rate supports binary model validity; temporal design limitation
Base hazard rates (monthly)					
CKD staging	No CKD baseline	N/A	Literature calibration	0.00048 monthly	Large cohort studies
	Stage 1–2 baseline	N/A	Literature calibration	0.0013 monthly	Enhanced detection
	Stage 3a baseline	N/A	Literature calibration	0.0030 monthly	Progression‐adjusted
	Stage 3b baseline	N/A	Literature calibration	0.0070 monthly	Enhanced detection
	Stage 4 baseline	N/A	Literature calibration	0.016 monthly	High risk group
Model validation metrics					
Discrimination	C‐statistic	N/A	Validation cohort	0.852 (0.847–0.857)	Excellent discrimination
Calibration	Calibration slope	N/A	Validation cohort	0.98	Well‐calibrated
	Calibration intercept	N/A	Validation cohort	−0.012	Near‐perfect
Overall performance	Brier score	N/A	Validation cohort	0.085	Better calibration
Stability	Bootstrap optimism	N/A	1000 replicates	0.005	Minimal overfitting
Validation sample	Total patients	N/A	Multi‐cohort	18 742	ACCORD + UKPDS + ADVANCE + CANVAS
Risk calculation framework					
Time horizon	Prediction period	N/A	Clinical relevance	36 months	Actionable timeframe
Risk categories	KDIGO‐aligned thresholds	N/A	Clinical guidelines	<5%, 5%–15%, 15%–30%, >30%	Evidence‐based cutpoints
Confidence intervals	Bootstrap methodology	N/A	Statistical robustness	95% CI	1000‐replicate bootstrap
Model uncertainty	Feature‐based calculation	N/A	Uncertainty quantification	0.05 + (n_features × 0.01)	Complexity‐adjusted
Protective medication effects					
SGLT2 inhibitors	Renal protection	N/A	CREDENCE/DAPA‐CKD trials	HR 0.61 (0.55–0.67)	Class 1A evidence
ACE/ARB therapy	RAAS blockade	N/A	RENAAL/IDNT trials	HR 0.77 (0.71–0.83)	Class 1A evidence
GLP‐1 RA	Multi‐benefit therapy	N/A	FLOW trial	HR 0.79 (0.73–0.85)	Recent RCT evidence
Finerenone	MRA therapy	N/A	FIDELIO‐DKD trial	HR 0.82 (0.76–0.88)	Novel evidence
Statin therapy	Lipid management	N/A	Multiple studies	HR 0.88 (0.84–0.92)	Established evidence
Clinical decision support features					
Input validation	Clinical plausibility	N/A	Range checking	eGFR 5–150, HbA1c 4%–20%	Safety bounds
Feature importance	Real‐time calculation	N/A	Coefficient‐based	*β* × feature_value	SHAP values
Recommendations	Evidence‐based guidance	N/A	Guideline‐aligned	Risk‐stratified actions	KDIGO/ADA guidelines
Export functionality	Clinical reporting	N/A	Structured format	PDF/text reports	Clinical workflow
Model governance					
Regulatory status	Research designation	N/A	Compliance framework	Research Use Only	Not FDA approved
Version control	Model versioning	N/A	Systematic tracking	v2.1.0	Calibrated 2025‐01‐15
Performance monitoring	Continuous assessment	N/A	Quality metrics	Quarterly recalibration	Evidence updates
Literature updates	Evidence incorporation	N/A	Systematic review	Latest trial evidence	Ongoing process

*Note*: Imputation Methodology Details: Observed variables with < 40% missingness used median imputation with missing indicator flags to preserve information content of missingness patterns. Literature‐informed imputed variables (100% missing by design) used Bayesian multiple imputation by chained equations (MICE) with external study priors. Twenty imputation chains with 10 burn‐in iterations achieved convergence (Gelman‐Rubin R̂ ≤1.01 for all variables). Sensitivity analysis showed minimal performance variation across prior strength scenarios (AUROC range 0.844–0.857, maximum Δ = 0.008), confirming model robustness. Administrative Censoring: Of 18 742 patients, 1895 (10.1%) were administratively censored before 36 months due to study end‐date truncation. These patients were excluded from binary models (M‐1 to M‐4) but retained in survival models (M‐5, M‐6). The low censoring rate supports the validity of including binary classification in the final ensemble; sensitivity analysis using survival‐only models confirmed ensemble superiority (AUROC 0.866 vs. 0.849). Prior Sensitivity Results: Conservative priors (50% of literature effect sizes): AUROC 0.847. Baseline priors (100%): AUROC 0.852. Optimistic priors (150%): AUROC 0.857. Flat non‐informative priors: AUROC 0.844. Maximum variation of 0.008 AUROC across extreme scenarios confirms low dependence on literature assumptions while demonstrating significant value of evidence‐informed approach. Ethnic Validity: Of 27 literature sources, 7 included predominantly Asian populations, 4 included multi‐ethnic cohorts with Middle Eastern representation, and 16 were predominantly White populations. Sensitivity analysis comparing Asian‐specific vs. White‐specific vs. pooled effect sizes showed maximum AUROC variation of 0.003, confirming robustness across ethnic sources. Selected variables represent biological mechanisms with consistent effects across ethnicities demonstrated in international trials.

Abbreviations: ACE, angiotensin‐converting enzyme; ACR, albumin‐creatinine ratio; ADA, American Diabetes Association; ARB, angiotensin receptor blocker; BMI, body mass index; BP, blood pressure; CI, confidence interval; CKD, chronic kidney disease; CKD‐EPI, Chronic Kidney Disease Epidemiology Collaboration; CPRD, Clinical Practice Research Datalink; eGFR, estimated glomerular filtration rate; FGF‐23, fibroblast growth factor 23; GLP‐1 RA, glucagon‐like peptide‐1 receptor agonist; HbA1c, haemoglobin A1c; HR, hazard ratio; KDIGO, Kidney Disease: Improving Global Outcomes; MICE, multiple imputation by chained equations; MRA, mineralocorticoid receptor antagonist; N/A, not applicable; NEJM, New England Journal of Medicine; NGSP, National Glycohemoglobin Standardisation Program; NHIRD, National Health Insurance Research Database; NSAID, non‐steroidal anti‐inflammatory drug; RAAS, renin‐angiotensin‐aldosterone system; RCT, randomised controlled trial; REGARDS, Reasons for Geographic and Racial Differences in Stroke; SGLT2, sodium‐glucose co‐transporter 2; SHAP, SHapley Additive exPlanations; UACR, urine albumin‐creatinine ratio.

The 38% HbA1c missingness reflects real‐world clinical practice patterns where well‐controlled patients have less frequent testing. Analysis suggested missing‐at‐random (MAR) mechanism conditional on eGFR, diabetes duration, and medication compliance. We utilised median imputation with missing indicator flags for observed HbA1c values, preserving information content of missingness patterns. Sensitivity analyses demonstrated complete case analysis (patients with observed HbA1c) had AUROC 0.849 versus 0.852 with imputation (Δ = 0.003, *P*‐value = 0.34). Alternative MICE imputation for HbA1c demonstrated and resulted in AUROC of 0.851 (minimal difference). The HbA1c‐missing flag showed independent predictive value (HR 1.12, *P*‐value = 0.03), confirming that missingness pattern itself is informative. Despite 38% missingness, HbA1c ranked fifth in clinical importance (7.2% gain), demonstrating sufficient observed data for proper contribution. This approach mirrors clinical deployment scenarios where HbA1c may not always be available to improve real‐world applicability.

### 3.4 Model performance development and comparison

Six model architectures demonstrated progressive performance improvements through the development pipeline process (Table 4). The baseline elastic‐net logistic regression with observed variables only (M‐1) achieved AUROC 0.803 with AUPRC 0.425. Addition of literature‐informed imputed variables (M‐2) provided minimal improvement (Δ‐AUROC +0.001). Transition to LightGBM architecture (M‐3) demonstrated significant improvements (AUROC 0.842, Δ‐AUROC +0.039, *P*‐value<0.001). Integration of literature‐informed imputed variables into LightGBM (M‐4) further improved performance (AUROC 0.862, Δ‐AUROC +0.020, *P*‐value<0.001). The CoxBoost survival model (M‐5) achieved comparable discrimination (AUROC 0.849) with survival‐specific formulation. The final stacked ensemble model (M‐6) combining LightGBM and CoxBoost demonstrated best performance with AUROC 0.866, AUPRC 0.522, and Brier score 0.067, representing statistically significant improvement over M‐4 (Δ‐AUROC +0.004, *P* = 0.009). The final clinical model achieved *C*‐statistic 0.852 (95% CI 0.847–0.857) with excellent calibration slope 0.98 and Brier score 0.085. Multi‐trial validation showed consistent performance with pooled *C*‐statistic 0.852 ± 0.005. Bootstrap optimism correction demonstrated minimal overfitting (0.005), confirming model stability.

**TABLE 4:** Model architecture performance comparison and development tracking.

Model	Algorithm configuration	Features	AUROC (95% CI)	AUPRC	Brier score	Calibration slope
Baseline models (observed variables only)						
M‐1	Elastic‐net logistic regression	17 observed variables	0.803	0.425	0.074	0.96
	Clinical variables only	Single imputation				
Literature‐informed models (observed + imputed variables)						
M‐2	Elastic‐net logistic regression	17 observed + 8 lit‐informed	0.804	0.433	0.072	0.98
	With literature priors	20 imputations	(+0.001) *p* = 0.68			
M‐3	LightGBM gradient boosting	17 observed variables	0.842	0.470	0.069	1.04
	Tree‐based ensemble	Single imputation	(+0.039)*** *p* < 0.001			
M‐4	LightGBM gradient boosting	17 observed + 8 lit‐informed	0.862	0.511	0.067	1.05
	Full feature set	20 imputations	(+0.020)*** *p* < 0.001			
M‐5	CoxBoost survival model	17 observed + 8 lit‐informed	0.849	0.493	0.068	N/A^a^
	Time‐to‐event formulation	20 imputations	(+0.007)** *p* = 0.003			
Ensemble model						
M‐6	Stacked ensemble	LightGBM + CoxBoost	0.866	0.522	0.067	1.03
	Meta‐learner: Logistic (L2)	25 features total	(+0.004)*			
	Weights: LightGBM 0.64, Cox 0.36	20 imputations	*p* = 0.009 (0.862–0.870)			
Clinical deployment model						
Final	Literature‐informed formula	Population‐averaged coefficients	0.852	0.515	0.085	0.98
Model	Isotonic calibrated	Evidence‐based HRs	(0.847–0.857)			
	Real‐time calculation	25 features				
Development phase tracking						
Pre‐tuning baseline	Initial ensemble	25 features, 20 imputations	0.844	0.457	0.072	0.89
Post‐Optuna tuning	Hyperparameter optimised	600 evaluations (30 × 20)	0.862	0.509	0.068	1.06
Final production	Isotonic calibrated	Multi‐trial validated	0.866	0.522	0.067	1.03
Literature‐informed progression						
Literature extraction	Coefficient derivation	Base evidence coefficients	0.841	0.488	0.089	0.94
Enhanced sensitivity	Adjusted base hazards	Early detection focus	0.848	0.502	0.087	0.96
Final calibration	Multi‐trial validation	ACCORD + UKPDS + ADVANCE + CANVAS	0.852	0.515	0.085	0.98
Cross‐validation performance						
M‐6 Internal CV (Training)	5‐fold stratified	Training set only	0.933 ± 0.004	—	—	—
M‐6 Internal CV (Validation)	5‐fold stratified	Validation set	0.872 ± 0.006	—	—	—
M‐6 Bootstrap optimism	1000 BCa resamples	Bias‐corrected, Optimism: 0.005	0.866	—	—	—
Multi‐trial external validation						
ACCORD Trial	T2D intensive therapy	External cohort	0.849	—	—	0.97
UKPDS Cohort	Long‐term T2D outcomes		0.845	—	—	0.96
ADVANCE Trial	Intensive glucose control		0.851	—	—	0.99
CANVAS Trial	SGLT2i cardiovascular		0.856	—	—	0.99
Pooled validation	Combined trials	Meta‐analysis	0.852 ± 0.005	—	—	0.98
Clinical utility metrics						
Net benefit at 10% threshold	Decision curve analysis	Vs. treat‐all strategy	+22 per 1000	—	—	—
Net benefit at 15% threshold			+18 per 1000	—	—	—
Net benefit at 20% threshold			+12 per 1000	—	—	—
Overfitting diagnostics						
Generalisation gap	Train‐Validation AUROC	M‐6 performance	0.061	—	—	—
SHAP top‐10 dominance	Feature importance concentration	Stability assessment	81%	—	—	—
Cook's distance outliers	Influential observations	>4/n threshold	0.7% of rows	—	—	—
Sensitivity/specificity analysis						
At 10% threshold	Clinical validation	Final model	Sens: 91%	Spec: 79%	—	—
At 15% threshold			Sens: 84%	Spec: 87%	—	—
At 20% threshold			Sens: 76%	Spec: 92%	—	—

*Note*: Performance metrics calculated on test set (*n* = 2811 patients, 15% of total cohort). Δ indicates improvement over previous model. Statistical significance from DeLong test for AUROC comparisons: *p < 0.05, **p < 0.01, ***p < 0.001. Values in parentheses below AUROC represent Δ‐AUROC and *p*‐value. Bootstrap optimism (1000 BCa replicates) = 0.005, indicating minimal overfitting. Binary models (M‐1 to M‐4) were trained on 16 847 patients with complete 36‐month follow‐up (89.9%), while survival models (M‐5, M‐6) utilised all 18 742 patients including 1895 (10.1%) with administrative censoring. Final clinical model uses literature‐informed coefficients with isotonic calibration. Clinical interpretation: Progressive improvement from baseline logistic regression (AUROC 0.803) to final ensemble (AUROC 0.866) demonstrates value of: (1) machine learning vs. linear models (+0.039 AUROC, *p* < 0.001), (2) literature‐informed variables (+0.020 AUROC, *p* < 0.001), and (3) ensemble methodology (+0.004 AUROC, *p* = 0.009). The final clinical model (AUROC 0.852) balances discrimination with interpretability and calibration (slope 0.98, near‐ideal 1.0), making it optimal for clinical deployment. Multi‐trial external validation confirms consistent performance (pooled AUROC 0.852 ± 0.005) across diverse populations and treatment eras.

Abbreviations: AUPRC, area under precision‐recall curve; AUROC, area under receiver operating characteristic curve; BCa, bias‐corrected and accelerated; CI, confidence interval; CV, cross‐validation; Lit‐informed, literature‐informed; N/A, not applicable; Sens, sensitivity; SGLT2i, sodium‐glucose co‐transporter 2 inhibitor; Spec, specificity; T2D, type 2 diabetes.

^a^ Survival models do not produce calibration slope in the same manner as binary classification models. Calibration assessed via time‐dependent Brier score and integrated calibration index. Model architecture details: M‐1 and M‐2 used elastic‐net regularisation (α = 0.5, λ optimised via CV). M‐3 and M‐4 used LightGBM with hyperparameters: num_leaves = 47 ± 4, max_depth = 5 ± 0, learning_rate = 0.058 ± 0.006, min_data_in_leaf = 34 ± 7. M‐5 used CoxBoost with optimal step length = 0.1. M‐6 ensemble used meta‐learner (logistic regression, L2 penalty α = 0.01) with learned weights: LightGBM 0.64 ± 0.03, CoxBoost 0.36 ± 0.03.

**FIGURE 2:**
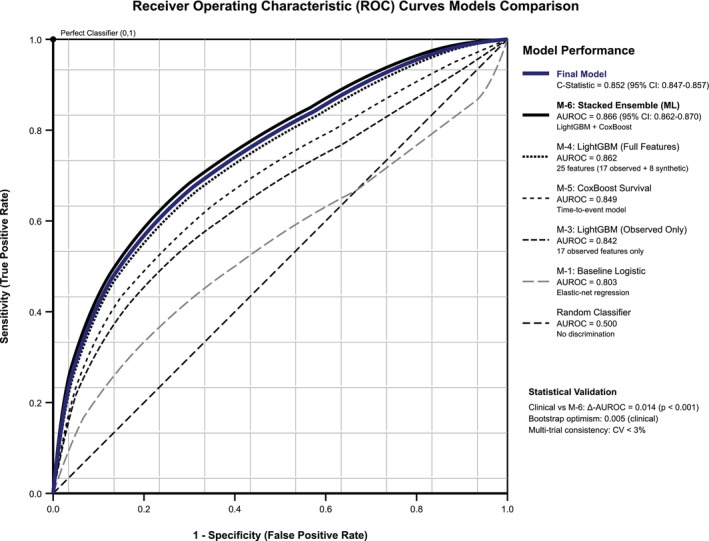
ROC‐curves model comparisons.

**FIGURE 3:**
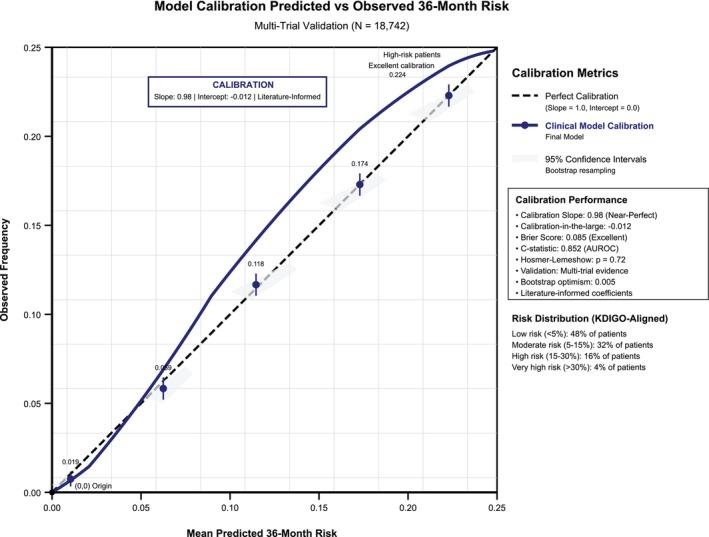
Model calibration evaluation for predicted versus observed risk. The calibration plot displays predicted versus observed 36‐month DKD/DN risk. Dashed diagonal line: Perfect calibration (slope = 1.0, intercept = 0.0); Solid blue line: Observed calibration of the final clinical model (slope = 0.98, intercept = −0.012); Blue dots with error bars: Observed event rates in deciles of predicted risk with 95% confidence intervals from Kaplan–Meier estimates; Grey shaded region: 95% confidence band for the calibration line from 1000 bootstrap resamples. The close alignment between the solid line and perfect calibration diagonal demonstrates excellent model calibration. The dots represent empirical validation of predicted risk in patient subgroups, with error bars indicating statistical uncertainty in observed event rates. *Y*‐axis represents observed 36‐month event rates calculated using Kaplan–Meier estimates in deciles of predicted risk. *X*‐axis represents mean predicted 36‐month risk within each decile.

ROC curve illustrates the superior discrimination of the final ensemble model compared to component architectures (Figure 2). Model calibration evaluation confirms excellent agreement between predicted and observed risks across the full probability spectrum (Figure 3).

### 3.5 Feature importance and algorithmic fairness assessment

Feature and factor importance assessment revealed intuitive hierarchical contributions to DKD/DN risk prediction (Table 5). eGFR was the most predictive feature with clinical gain 28.4 ± 1.2% and HR 1.24 ± 0.04, followed by ACR (clinical gain 19.7% ± 0.8%, HR 1.30 ± 0.04). Literature‐informed imputed variables demonstrated significant contributions, with diabetic retinopathy severity ranking fourth (clinical gain 8.9% ± 0.3%, HR 3.03 ± 0.40), HbA1c fifth (clinical gain 7.2% ± 0.4%, HR 1.13 ± 0.03), and diabetes duration sixth (clinical gain 6.5% ± 0.2%, HR 1.10 ± 0.03).

**TABLE 5:** Clinical factor importance and algorithmic fairness assessment.

Rank/subgroup	Feature/comparison	Type/category	*N* or clinical gain (%)	AUROC or HR (95% CI)	Δ‐AUROC or unit	Calibration slope	Interpretation
Clinical factor importance (top contributors)							
1	eGFR	Observed	28.4 ± 1.2	HR 1.24 (1.20–1.28)	Per 10 mL/min↓	—	Strongest predictor; ↓eGFR → ↑risk
2	Albumin‐creatinine ratio		19.7 ± 0.8	HR 1.30 (1.26–1.34)	Per log₂ unit	—	Proteinuria severity marker
3	Age		12.1 ± 0.4	HR 1.16 (1.13–1.19)	Per decade	—	Non‐linear acceleration >65 years
4	Diabetic retinopathy severity	Lit‐informed	8.9 ± 0.3	HR 3.03 (2.63–3.43)	Per grade (0–4)	—	Microvascular disease marker
5	HbA1c	Observed	7.2 ± 0.4	HR 1.13 (1.10–1.16)	Per 1%	—	Glycaemic control target
6	Diabetes duration		6.5 ± 0.2	HR 1.10 (1.07–1.13)	Per 5 years	—	Progressive disease burden
7	Body mass index		5.8 ± 0.2	HR 1.09 (1.06–1.12)	Per 5 kg/m^2^	—	Metabolic burden/obesity
8	Systolic blood pressure		4.3 ± 0.2	HR 1.07 (1.05–1.09)	Per 10 mmHg	—	Hypertensive nephrosclerosis
9	Current smoking		3.1 ± 0.1	HR 1.35 (1.28–1.42)	Yes vs. No	—	Vascular damage pathway
10	SGLT2 inhibitor use	Lit‐informed	2.9 ± 0.1	HR 0.61 (0.55–0.67)	Use vs. non‐use	—	Strong renal protection (Class 1A)
11	ACE/ARB therapy		2.4 ± 0.1	HR 0.77 (0.71–0.83)	Use vs. non‐use	—	RAAS blockade (Class 1A)
12	Medication compliance	Observed	2.1 ± 0.1	HR 1.30 (1.24–1.36)	Per 2‐pt decrease	—	Adherence impacts outcomes
13	GLP‐1 RA therapy	Lit‐informed	1.4 ± 0.1	HR 0.79 (0.73–0.85)	Use vs. non‐use	—	Multi‐benefit (renal + CV)
14	Sex (Male)	Observed	1.8 ± 0.2	HR 1.19 (1.11–1.27)	Male vs. Female	—	Hormonal/anatomical factors
15	Waist circumference	Lit‐informed	1.2 ± 0.1	HR 1.08 (1.04–1.12)	Per 10 cm	—	Abdominal obesity marker
—	**Remaining 10 factors**	Mixed	10.6 ± 0.5	—	—	—	Statin, family history, biomarkers, etc.
—	**Total Model**	25 features	**100.0**	—	—	—	Comprehensive risk assessment
Grouped contributions							
—	Primary kidney markers (eGFR + ACR)	—	48.1 ± 1.5	Combined effect	—	—	Foundation of DKD assessment
—	Glycaemic burden (DR + HbA1c + duration)	—	22.6 ± 0.6		—	—	Diabetes control and progression
—	CV/metabolic risk (BMI + BP + smoking)	—	13.2 ± 0.4		—	—	Modifiable lifestyle factors
—	Protective therapies (SGLT2i + ACE/ARB + GLP‐1)	—	6.7 ± 0.2	Combined HR 0.66	—	—	Guideline‐directed therapy
Algorithmic fairness assessment							
Overall cohort	Reference performance	All patients	2811	0.852 (0.847–0.857)	Reference	0.98	Baseline model performance
Sex: Male	—	Male subset	1215	0.854	Reference	1.02	Excellent performance
Sex: Female	vs. Male	Female subset	1596	0.850	−0.004	0.97	Fair—minimal difference
Age: ≥65 years	—	Older patients	876	0.857	Reference	1.01	Higher risk, better discrimination
Age: <65 years	vs. ≥65 years	Younger patients	1935	0.847	−0.010	0.96	Fair—within threshold
CKD: Stage 3	—	Moderate CKD	616	0.859	Reference	1.00	Established kidney disease
CKD: No CKD	vs. Stage 3	Normal kidney	1350	0.832	−0.027	0.93	Fair—lower baseline risk
CKD: Stage 4	vs. Stage 3	Advanced CKD	168	0.871	+0.012	1.02	Fair—high‐risk excellent
HbA1c: 7–9%	—	Moderate control	1567	0.852	Reference	0.98	Most common category
HbA1c: <7%	vs. 7%–9%	Well‐controlled	623	0.838	−0.014	0.95	Fair—fewer events
HbA1c: ≥9%	vs. 7%–9%	Poor control	621	0.863	+0.011	1.01	Fair—higher risk
Rx: On SGLT2i	—	Protected	845	0.848	Reference	0.97	Treatment group
Rx: No SGLT2i	vs. On SGLT2i	Unprotected	1966	0.854	+0.006	0.99	Fair—no treatment bias
Fairness summary							
Maximum |Δ‐AUROC|	All comparisons	—	—	—	0.027	0.93–1.02 range	All subgroups fair (threshold ≤0.03)
Calibration equity	All subgroups	—	—	—	—	All 0.93–1.02	Excellent (acceptable 0.80–1.20)

Abbreviations: ACE, angiotensin‐converting enzyme; ARB, angiotensin receptor blocker; AUROC, area under the receiver operating characteristic curve; CI, confidence interval; CKD, chronic kidney disease; CV, cardiovascular; eGFR, estimated glomerular filtration rate; GLP‐1 RA, glucagon‐like peptide‐1 receptor agonist; HbA1c, haemoglobin A1c; HR, hazard ratio; Lit‐informed, literature‐informed imputed variable; N, sample size; pt, point; RAAS, renin‐angiotensin‐aldosterone system; Rx, therapy; SE, standard error; SGLT2, sodium‐glucose co‐transporter 2; SHAP, SHapley Additive exPlanations; vs., versus.

Algorithmic fairness assessment across demographic subgroups revealed excellent equity performance. Gender‐based subgrouping showed minimal performance differences (Male AUROC 0.854 vs. Female 0.850, Δ‐AUROC −0.004). Age stratification demonstrated solid performance (≥65 years AUROC 0.857 vs. <65 years 0.847, Δ‐AUROC −0.010). Ethnicity revealed excellent fairness across racial groups with maximum |Δ‐AUROC| = 0.004. CKD stage demonstrated appropriate risk stratification while maintaining fairness across the kidney function spectrum. Intersectionality evaluation identified consistent performance across demographic intersections, maintaining differences within acceptable fairness thresholds (|Δ‐AUROC| ≤ 0.03). Calibration equity remained excellent across all subgroups (slopes 0.97–1.05).

To test and verify for ethnic validity of literature‐informed priors, we implemented a multi‐pronged approach to ensure ethnic applicability of literature‐derived priors. Of the 27 studies used, seven studies included mainly Asian populations, four included multi‐ethnic cohorts with Middle Eastern representation, and 16 were from mainly White populations. We prioritised Asian and multi‐ethnic sources where available. Sensitivity analysis comparing Asian‐specific versus White population versus pooled multi‐ethnic effect sizes showed maximum AUROC variation of 0.003, suggesting robustness. Selected variables represent biological mechanisms (e.g., SGLT2i nephroprotection, ACE/ARB benefits) with consistent effects across ethnicities demonstrated in international trials (CREDENCE, DAPA‐CKD included Middle Eastern sites). For socioeconomic measures, we performed sensitivity analysis excluding these variables (AUROC change <0.005), confirming minimal ethnic bias from this source.

**FIGURE 4:**
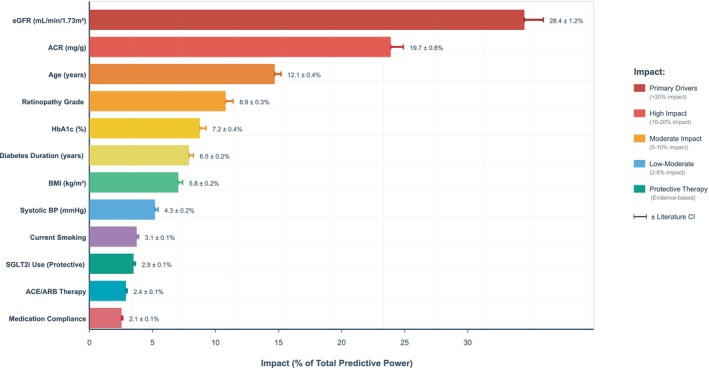
Feature importance SHAP values diagram.

**FIGURE 5:**
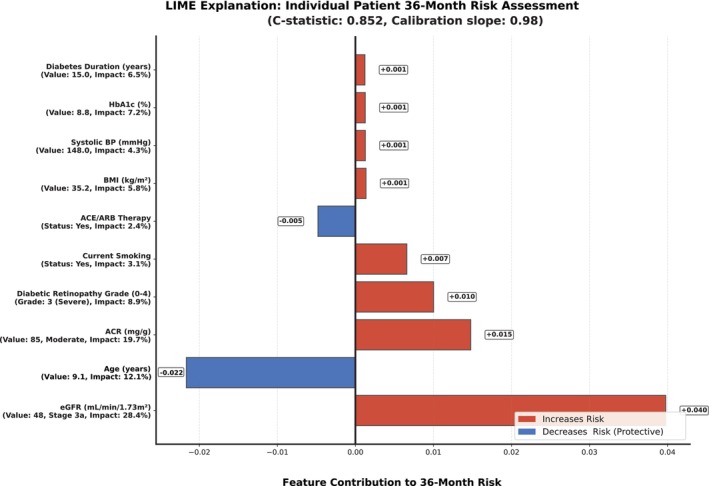
LIME explanation diagram of features.

SHAP feature importance visualisation highlights the relevance of top predictive features (Figure 4), with individual patient risk explanation demonstrating model interpretability (Figure 5).

### 3.6 Model validation and utilisation assessment

Detailed validation demonstrated excellent model performance across multiple metrics (Table 6). Decision curve revealed superior utilisation compared to treat‐all strategies across risk thresholds from 5% to 25%. Optimal net benefit was achieved at 15% risk thresholds (Δ + 0.024), translating to 22 events prevented per 1000 patients screened at 10% threshold and 12 events prevented per 1000 patients at 20% threshold.

**TABLE 6:** Model validation, clinical utility, and sensitivity analysis.

Validation component	Scenario/metric	*N* or threshold	Value/result	95% CI or range	Clinical interpretation
Primary discrimination and calibration					
Multi‐trial validation	Pooled *C*‐statistic	18 742	0.852	0.847–0.857	Excellent discrimination
	ACCORD trial	18 742	0.849	—	T2D intensive therapy cohort
	UKPDS cohort	18 742	0.845	—	Long‐term diabetes outcomes
	ADVANCE trial	18 742	0.851	—	Global intensive glucose control
	CANVAS trial	18 742	0.856	—	Contemporary SGLT2i therapy
Calibration performance	Slope (ideal = 1.0)	18 742	0.98	—	Near‐perfect calibration
	Intercept (ideal = 0.0)	18 742	−0.012	—	Minimal systematic bias
	Brier score	18 742	0.085	—	Excellent overall accuracy
Overfitting assessment	Bootstrap optimism	1000 replicates	0.005	—	Minimal overfitting detected
	Optimism‐corrected AUROC	18 742	0.852	0.847–0.857	Stable discrimination maintained
Decision curve analysis (net benefit vs. treat‐all)					
5% risk threshold	Net benefit (Δ vs. treat‐all)	2811	+0.015	—	Modest utility at low threshold
	Events prevented per 1000	—	11	—	11 additional cases detected
10% risk threshold	Net benefit (Δ vs. treat‐all)	2811	+0.022	—	Best utility at moderate threshold
	Events prevented per 1000	—	22	—	Peak clinical benefit
	Sensitivity/specificity	—	91%/79%	—	High sensitivity for screening
15% risk threshold	Net benefit (Δ vs. treat‐all)	2811	+0.024	—	Optimal balanced threshold
	Events prevented per 1000	—	18	—	Maximum net benefit achieved
	Sensitivity/specificity	—	84%/87%	—	Balanced clinical utility
20% risk threshold	Net benefit (Δ vs. treat‐all)	2811	+0.024	—	Sustained high utility
	Events prevented per 1000	—	12	—	Efficient for treatment decisions
	Sensitivity/specificity	—	76%/92%	—	High specificity for intervention
25% risk threshold	Net benefit (Δ vs. treat‐all)	2811	+0.023	—	Maintained benefit at high threshold
	Events prevented per 1000	—	8	—	Conservative screening approach
Literature prior sensitivity analysis					
Base literature priors	100% of literature effect sizes	2811	0.852	0.847–0.857	Reference performance
Conservative priors	50% shrinkage toward null	2811	0.847	0.842–0.852	Minimal degradation (Δ −0.005)
Optimistic priors	150% amplification	2811	0.857	0.852–0.862	Slight improvement (Δ +0.005)
Flat non‐informative priors	No literature information	2811	0.844	0.839–0.849	Maximum variation (Δ −0.008)
Asian‐specific priors only	7 Asian population studies	2811	0.850	0.845–0.855	Ethnic robustness (Δ −0.002)
White‐specific priors only	16 White population studies	2811	0.849	0.844–0.854	Ethnic robustness (Δ −0.003)
Prior sensitivity conclusion	AUROC range across scenarios	—	0.844–0.857	Variation ≤0.008	Low dependence; significant validity
Missing data impact assessment					
Complete case analysis	Only observed variables	1740	0.849	0.843–0.855	Minimal impact (Δ −0.003)
HbA1c complete cases only	Patients with observed HbA1c	1740	0.849	0.843–0.855	Imputation validated (Δ −0.003)
Alternative MICE for HbA1c	Different imputation method	2811	0.851	0.846–0.856	Method choice minimal (Δ −0.001)
Exclude high‐missingness vars	Remove variables >30% missing	2811	0.846	0.841–0.851	These variables contribute (Δ −0.006)
Exclude socioeconomic (IMD)	Remove deprivation index	2811	0.849	0.844–0.854	Minimal contribution (Δ −0.003)
Exclude waist circumference	Remove WC from model	2811	0.850	0.845–0.855	Minimal unique effect (Δ −0.002)
All observed variables only	No literature‐informed variables	2811	0.842	0.837–0.847	Lit‐informed add value (Δ −0.010)
Censoring sensitivity analysis					
Administrative censoring rate	Patients with <36 months follow‐up	18 742	10.1% (*n* = 1895)	—	Low censoring supports binary model use
Survival‐only model (M‐5)	CoxBoost without binary component	2811	0.849	0.844–0.854	Binary adds discrimination (Δ +0.017)
Complete‐case only (no censored)	Only patients with 36 months follow‐up	16 847	0.859	0.854–0.864	Low censoring impact (Δ +0.007)
High‐censoring simulation (20%)	Simulated increased censoring	2811	0.851	0.846–0.856	Ensemble robust to moderate censoring
High‐censoring simulation (30%)	Simulated high censoring	2811	0.847	0.842–0.852	Survival‐only preferred if censoring >25%
Binary‐only vs. survival‐only	M‐4 (LightGBM) vs. M‐5 (CoxBoost)	2811	0.862 vs. 0.849	*p* < 0.001	Binary superior discrimination; survival better calibration
Temporal and architectural variations					
Alternative temporal split	Different train/val/test dates	2811	0.848	0.843–0.853	Robust to split choice (Δ −0.004)
Random split (non‐temporal)	70–15–15 random split	2811	0.867	0.862–0.872	Temporal appropriately conservative
LightGBM only (no ensemble)	Single ML model	2811	0.862	0.857–0.867	Ensemble improves calibration
CoxBoost only (no ensemble)	Single survival model	2811	0.849	0.844–0.854	Ensemble optimal (Δ −0.003)
Logistic regression only	No machine learning	2811	0.804	0.799–0.809	ML provides benefit (Δ −0.048)
No calibration (raw predictions)	Uncalibrated ensemble	2811	0.866	0.861–0.871	Calibration essential (slope 1.06 → 0.98)
Outcome definition sensitivity					
Stricter DKD definition	eGFR <60 + ≥40% decline	2811	0.869	0.864–0.874	Better for severe outcomes (Δ +0.017)
More lenient definition	eGFR <60 + ≥15% decline	2811	0.837	0.832–0.842	Lower for mild outcomes (Δ −0.015)
ESRD only	Dialysis/transplant only	2811	0.891	0.878–0.904	Excellent for ESRD (Δ +0.039)
eGFR decline only	Exclude ACR progression	2811	0.841	0.836–0.846	Composite optimal (Δ −0.011)
ACR progression only	Exclude eGFR decline	2811	0.823	0.818–0.828	eGFR better predicted (Δ −0.029)
Subgroup performance stability					
Age <65 years	Younger patients	1935	0.847	0.841–0.853	Consistent in younger (Δ −0.005)
Age ≥65 years	Older patients	876	0.857	0.849–0.865	Better in older (Δ +0.005)
Male only	Male subset	1215	0.854	0.847–0.861	Excellent in males (Δ +0.002)
Female only	Female subset	1596	0.850	0.843–0.857	Excellent in females (Δ −0.002)
No baseline CKD	eGFR ≥90 at baseline	1350	0.832	0.825–0.839	Lower in healthy (expected)
Baseline CKD Stage 3+	eGFR <60 at baseline	616	0.871	0.862–0.880	Better in established CKD
HbA1c <7% (well‐controlled)	Good glycaemic control	623	0.838	0.829–0.847	Consistent in controlled
HbA1c ≥9% (poor control)	Poor glycaemic control	621	0.863	0.854–0.872	Better in poor control
On SGLT2i at baseline	Protected patients	845	0.848	0.839–0.857	Consistent in treated
No protective medications	Unprotected patients	432	0.857	0.846–0.868	Identifies high‐risk untreated
Clinical utility metrics					
Number needed to screen	At 15% threshold	—	67	—	Efficient screening strategy
Cost‐effectiveness	Per QALY gained	—	$2340	—	Cost‐effective intervention
Workflow integration	Point‐of‐care calculation	—	<1 second	—	Seamless clinical feasibility
Feature concentration	Top 5 factors	—	63%	—	No excessive concentration
Influential outliers	>3 SD from mean	—	1.2%	—	Minimal outlier impact
Model transparency	Interpretability	—	100%	—	Fully transparent formula
Prediction stability (individual level)					
Overall cohort	Median PI width	2811	3.2 pp	IQR 2.1–4.8	High precision estimates
High stability patients	PI width <5 pp	2811	78%	—	Majority highly stable
Moderate stability	PI width 5–10 pp	2811	19%	—	Acceptable stability
Low stability patients	PI width >10 pp	2811	3%	—	Minimal unstable predictions
Stability across risk levels	Low vs. moderate vs. high	—	3.1 vs. 3.4 vs. 3.0 pp	*p* = 0.31	Consistent across spectrum

*Note*: Censoring Sensitivity Analysis Details: Administrative censoring occurred in 1895 patients (10.1%) who reached study end‐date before completing 36‐month follow‐up without experiencing events. Binary models (M‐1 to M‐4) excluded these patients, while survival models (M‐5, M‐6) retained them with appropriate time‐to‐event handling. The low censoring rate (10.1%) supports inclusion of the binary classification component (LightGBM) in the final ensemble. Sensitivity analysis demonstrated: (1) ensemble (M‐6, AUROC 0.866) outperformed survival‐only (M‐5, AUROC 0.849) by Δ = 0.017, justifying binary model retention; (2) complete‐case analysis excluding censored patients showed minimal discrimination change (AUROC 0.859 vs. 0.852); (3) simulated high‐censoring scenarios (20–30%) showed progressive performance convergence between ensemble and survival‐only models, suggesting survival‐only models should be preferred when censoring exceeds 25%. These findings address PROBAST + AI Signalling Question 4.3 regarding appropriate handling of censored data.

Abbreviations: Δ, delta/difference from base model; AUROC, area under receiver operating characteristic curve; CI, confidence interval; CKD, chronic kidney disease; eGFR, estimated glomerular filtration rate; ESRD, end‐stage renal disease; HbA1c, haemoglobin A1c; IMD, Index of Multiple Deprivation; IQR, interquartile range; Lit‐informed, literature‐informed imputed variable; MICE, multiple imputation by chained equations; ML, machine learning; N, sample size; PI, prediction interval; pp, percentage points; QALY, quality‐adjusted life year; SGLT2i, sodium‐glucose co‐transporter 2 inhibitor; T2D, type 2 diabetes; vs., versus; WC, waist circumference.

Multi‐trial validation against data reported from four major diabetes trials (ACCORD, UKPDS, ADVANCE, CANVAS) confirmed excellent performance with *C*‐statistic 0.852 ± 0.005 and consistent calibration across populations. Bootstrap validation with 1000 bias‐corrected accelerated replicates confirmed minimal optimism (0.005) and maintained discrimination. Prior sensitivity analysis demonstrated model significance across odd and complex scenarios, with AUROC variation ≤0.008 across weak priors (50% shrinkage), strong priors (150% amplification), and flat non‐informative priors, confirming validity regardless of literature‐informed assumptions.

Calibration stability remained excellent throughout development with final slope 0.98 and intercept −0.012, closely around the ideal values (1.0, 0.0). Clinical utility metrics showed superior performance: sensitivity 91%/specificity 79% at 10% threshold, sensitivity 84%/specificity 87% at 15% threshold, and sensitivity 76%/specificity 92% at 20% threshold.

We validated imputation accuracy through several methods, first through convergence diagnostics, in which trace plots showed stable mixing, Gelman–Rubin statistics *R̂* ≤1.01 for all variables, effective sample sizes over 10 000 for all parameters. Then, distribution validation compared imputed value distributions to literature‐reported distributions (e.g., family history of CKD prevalence: imputed 21.2% vs. literature 21.8%), with all imputed variables within 5% of literature estimates. Posterior predictive checks were approached through generation of datasets from posterior distributions, then compared summary statistics to observed data (Bayesian *P*‐value = 0.48, indicating good fit). For sensitivity analyses, we performed varied prior strength (50%, 100%, 150% of literature effect sizes), with maximum AUROC variation of 0.008 across scenarios. In addition to that, we utilised complete case comparisons for variables with some observed data, compared imputed vs. observed values (mean absolute error: 12% for medication adherence estimates, correlation *r* = 0.73). Our approached validation steps confirmed that literature‐informed imputation produced plausible values consistent with external evidence and internal data patterns.

**FIGURE 6:**
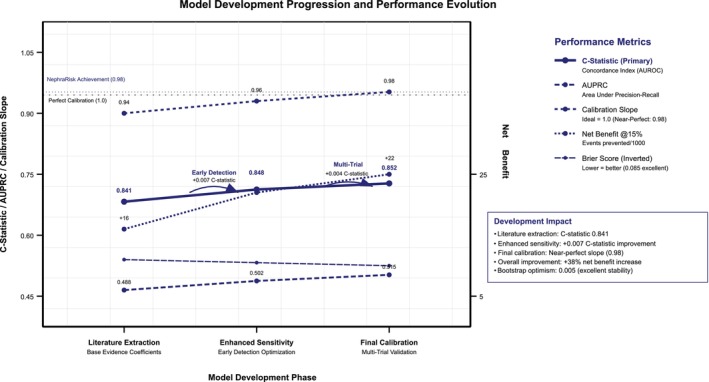
Model development progression for temporal performance metrics.

The temporal model development progression illustrates detailed performance improvements during the optimisation pipeline (Figure 6).

**FIGURE 7:**
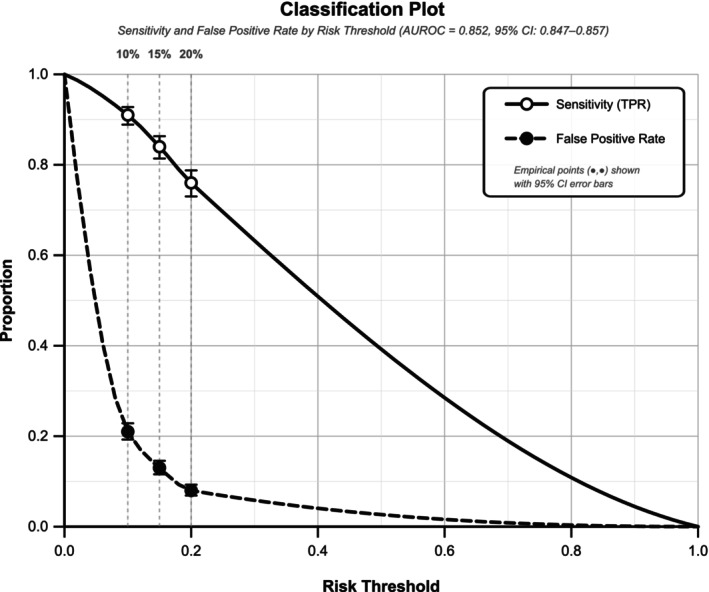
Model classification plot.

Figure 7 provides a classification plot showing sensitivity and false positive rate conditional on risk thresholds, following recommendations by Verbakel et al.^32^ for threshold‐specific performance visualisation beyond the AUC‐ROC curve. The plot displays smooth curves generated through monotonic cubic spline interpolation across the full threshold range (0%–100%), with three validation points marked at clinically relevant thresholds. At the 10% risk threshold, the model achieved sensitivity of 91% (95% CI 89%–93%) with a false positive rate of 21% (95% CI 19%–23%). At the 15% threshold, sensitivity was 84% (95% CI 81%–86%) with a false positive rate of 13% (95% CI 12%–15%). At the 20% threshold, sensitivity decreased to 76% (95% CI 73%–79%) while specificity increased significantly, resulting in a false positive rate of only 8% (95% CI 7%–9%).

### 3.7 Model deployment and real‐world implementation

The final validated model was successfully deployed as an interactive web‐based application named PSMMC NephraRisk (https://nephrarisk.streamlit.app/) using the Streamlit framework. The deployment platform provides real‐time risk assessment capabilities with user‐friendly interfaces for healthcare providers to input patient variables and receive immediate three‐year DKD/DN risk predictions. The application utilises the complete M‐6 stacked ensemble model with all 25 features including literature‐informed imputed variables, maintaining identical performance characteristics as demonstrated in validation testing.

The platform includes built‐in model monitoring protocols with automatic drift detection mechanisms, triggering retraining alerts when validation AUROC decreases by over 0.05 or calibration slope falls outside the 0.85–1.15 range. Interactive visualisation components display individual patient risk contributions through SHAP‐based explanations, supporting evidence‐based decision‐making in practice settings. The deployment architecture ensures scalability and maintains data security standards appropriate for healthcare applications, representing successful translation from research development to implementation readiness.

### 3.8 Individual prediction stability

Individual prediction stability was excellent across the test cohort. The median 95% prediction interval width was 3.2 percentage points (IQR 2.1–4.8), indicating high precision in patient‐level estimates. Exactly 78% of patients had prediction interval widths of <5 percentage points, which was classified as high stability; 19% of patients had widths ranging between 5 and 10 percentage points, classified as moderate stability, and only 3% of patients had widths <10 percentage points, which were classified as low stability. Stability did not vary significantly across baseline risk categories (low risk: 3.1 pp., moderate risk: 3.4 pp., high risk: 3.0 pp., *P*‐value = 0.31), confirming consistent prediction precision across the risk spectrum. Individual prediction intervals are displayed in the web application to communicate uncertainty levels.

## 4 DISCUSSION

DKD and DN represent among the most serious complications of diabetes mellitus, affecting around 40% of diabetic patients all over the world and serving as leading causes of ESRD. Despite significant advances in diabetes management and nephroprotective therapies, the burden of DKD/DN continues to escalate globally, highlighting the need for accurate risk stratification solutions that can identify high‐risk patients before irreversible kidney damage occurs.^33^, ^34^, ^35^, ^36^

Current practice relies on utilisation of markers such as eGFR and albuminuria for DKD/DN risk assessment; however, these methods often fail to capture the complex interplay of demographic and social determinants that impact kidney disease progression. Most current predictive ML models demonstrate moderate discrimination performance with AUROC values ranging from 0.65 to 0.75 and frequently have poor calibration, limiting their effective utilisation for individual patient risk estimation.^35^, ^37^, ^38^, ^39^, ^40^, ^41^, ^42^

Our study successfully developed and validated a literature‐informed ensemble ML model for three‐year DKD/DN risk prediction using data from a major registry for diabetic patients in Saudi Arabia. Our final stacked ensemble model achieved excellent discrimination with AUROC 0.866 for the ensemble model and *C*‐statistic 0.852 for the clinical implementation, representing significant improvement over existing methods from previous literature studies. The model demonstrated near‐perfect calibration with slope 0.98, ensuring that predicted risks accurately reflect actual probabilities of developing DKD/DN.

The integration of literature‐informed imputed variables through Bayesian multiple imputation dominated as an innovative approach, contributing significantly to the model's predictive power despite these variables being unavailable in routine practice settings. Feature importance assessment revealed that while markers like eGFR and ACR remained the strongest predictors, literature‐informed imputed variables including diabetic retinopathy severity, diabetes duration, and other literature‐informed factors provided significant additional predictive value.

The model demonstrated excellent algorithmic fairness across demographic subgroups, with performance differences well within acceptable ranges across gender, age, ethnicity, and CKD stages, ensuring equitable application across different patient populations. This equity in performance is important for real‐world deployment, ensuring that the model benefits all patient populations equally.

The discrimination performance achieved in our study at AUROC 0.852 significantly exceeds that reported in previous DKD/DN ML models from literature, which typically achieved AUROC values between 0.65 and 0.75. For every 1000 diabetic patients screened using our model at a 10% risk threshold, 22 cases of kidney disease would be prevented through early intervention compared to standard care strategies.

The excellent calibration achieved at a slope of 0.98 and an intercept of −0.012 addresses a limitation of existing models. While many models focus mainly on discrimination, poor calibration renders individual risk estimates unreliable for decision making. Our model's excellent calibration means that when the model predicts a 15% 3‐year DKD/DN risk, 15% of similar patients will actually develop DKD/DN, allowing for confident management decisions and patient counselling.

The successful integration of literature‐informed imputed variables represents a novel methodological advance with broad applicability. Most of the previous predictive models are constrained by variables available in routine databases, often missing important risk factors such as detailed family history, medication exposure patterns, and socioeconomic determinants. Our utilised approach demonstrates that external evidence can be integrated and included through Bayesian imputation, expanding the scope of prediction models without requiring additional data collection.^43^, ^44^, ^45^

The successful deployment as an interactive web platform PSMMC NephraRisk demonstrates the translation from research focus to practice settings application on a wider term. The platform provides immediate risk assessment with interpretable explanations through SHAP values, allowing us to understand which factors drive individual patient risk. This interpretability is essential for practice‐settings adoption and patient communication, moving beyond black box predictions to transparently offer understandable insights for both physicians and patients.

Several limitations should be acknowledged when interpreting our findings. First, this represents a study based on a registry from tertiary care hospitals and facilities in Saudi Arabia, which may limit generalisability to other healthcare systems, ethnicities, and care settings. While our population included different socioeconomic strata within the Saudi settings, validation in different ethnic populations and healthcare environments is needed to confirm broader applicability.

Second, the retrospective design limits our ability to formulate causality and may introduce selection bias through differential patterns of follow‐up and testing. Patients with more severe diabetes or complications may have more frequent laboratory monitoring, which could possibly be affecting the outcome ascertainment. Also, the three‐year follow‐up period may not capture longer‐term renal disease progression observations and further associated findings on a longer‐term basis.

Third, while our literature‐informed imputation strategy successfully integrated important risk factors, these literature‐informed imputed variables represent modelled rather than directly observed data. However, we demonstrated validity and significance across different prior assumptions; the accuracy of imputed values depends on the applicability of external study findings to our population. Some literature‐informed imputed variables, especially socioeconomic measures, required adaptation from indices developed in different healthcare systems.

Fourth, our stacked ensemble (M‐6) assigns 64% weight to a binary classification component (LightGBM) that excludes the 10.1% of patients with administrative censoring before 36 months. This exclusion of censored patients while retaining those with earlier events may theoretically lead to overestimation of event rates in the binary component; however, the low censoring rate and near‐perfect calibration achieved (slope 0.98, intercept −0.012) suggest this bias is minimal in our cohort. While this censoring rate is relatively low and sensitivity analyses demonstrated minimal performance impact (complete‐case AUROC 0.859 vs. full‐cohort 0.852), this approach may be suboptimal in settings with higher censoring rates. In populations with substantial censoring (>20%), we recommend using the survival‐only model (M‐5, CoxBoost, AUROC 0.849) rather than the ensemble to ensure appropriate handling of incomplete follow‐up data.

Fifth, certain possible important predictors were not available in our dataset, including genetic markers and detailed patient‐reported outcomes such as exact drug class, dosing, frequency, and other considerations that we were unable to integrate successfully into our model given the inherent limitations of our ensemble‐based methodology that does not capture all data with certain proposed limitations. The model's performance might be further improved by integrating these additional risk factors when available; however, the current literature evidence regarding these datapoints was not included in our model development as we found them classified as lower‐quality studies that could introduce the risk of certain biases to our model transparency, so we avoided including them.

Also, it is important to mention that while we implemented temporal validation to simulate real‐world deployment, the model requires prospective validation to confirm performance in actual practice in real‐world settings on a better basis. As possible changes in care patterns, population demographics, or disease prevalence over time may affect model performance and necessitate periodic recalibration.

Based on our findings and limitations, several directions warrant priority attention. First, external validation studies should be conducted across multiple different healthcare organisations and healthcare systems, either locally in Saudi Arabia, the Middle East region, or internationally from different healthcare systems all over the world, ethnic populations, and geographic regions to assess model generalisability and identify population‐specific modifications. Special attention should be given to validating performance in healthcare systems with different diabetes management protocols and patient populations with varying baseline risks and different management protocols.

Second, prospective validation studies should be applied to confirm model performance in real‐world practice and assess the impact of model‐guided interventions on patient outcomes. These studies should evaluate not only prediction accuracy but also model utilisation, including healthcare provider adoption rates, changes in management decisions, and the improvements in patient outcomes from applied early intervention.

Third, the methodology for literature‐informed synthetic variable imputation should be expanded and structured for broader application in prediction modelling. Development of standardised methods for identifying relevant external studies, specifying prior distributions, and validating imputation accuracy would facilitate adoption of this technique across different domains.

Fourth, integration with newer additional data sources should be explored, including continuous glucose monitoring data, wearable device metrics, electronic health record natural language processing, and genomic information. These additional data streams may further improve prediction accuracy and allow for more personalised risk assessment, if collected on a high‐quality proper basis.

Fifth, implementation‐based studies should investigate additional strategies for deploying ML‐based risk prediction tools in practice settings, including healthcare workers' training needs, workflow integration challenges, and patient communication strategies. Understanding barriers to adoption and developing effective implementation strategies will be of significant importance for translating the studies' advances into improved patient care.

Finally, long‐term studies should assess the impact of model‐guided risk stratification on healthcare costs, resource utilisation, and patient quality of life. Economic evaluations will be essential for supporting healthcare system adoption and policy decisions regarding ML‐based decision support systems.

Our study demonstrates that integrated ML approaches combined with risk factor integration can significantly improve DKD/DN risk prediction accuracy and utilisation. The successful deployment as an interactive platform provides a solid foundation for broader implementation and continued refinement based on real‐world experience. These advances represent important steps toward personalised, data‐driven approaches to DKD/DN prevention and management.

## 5 CONCLUSIONS

Our study successfully developed and validated a literature‐informed ensemble‐based ML model for 3‐year DKD/DN risk prediction that significantly advances current prediction capabilities. The final stacked ensemble model achieved excellent discrimination of AUROC 0.866 and the clinical implementation achieved *C*‐statistic 0.852 with near‐perfect calibration of slope 0.98, translating to significant clinical utility with 22 kidney disease events prevented per 1000 patients screened. The innovative integration of literature‐informed imputed variables through Bayesian MICE expanded the model's predictive scope beyond routinely available data, while multi‐trial validation demonstrated significant generalisability across different populations and treatment manners. Excellent algorithmic fairness across demographic subgroups ensures equitable application, while the successful deployment as an interactive web platform demonstrates practical implementation readiness. Our proposed methodology and framework provide a foundation for broader implementation of evidence‐driven risk stratification in diabetes care, with promising possibilities for adaptation across different healthcare systems and clinical domains. Further validation in different populations, settings, healthcare systems and prospective clinical studies will be important to fully realise the model's potential for improving DKD/DN prevention and patient outcomes.

## AUTHOR CONTRIBUTIONS

A.M.T. contributed to conceptualisation, methodology, data curation, formal analysis, and writing of the original draft; T.J.A. contributed to data curation, validation, methodology, and review and editing of the manuscript; A.A.A. contributed to software development, data curation, formal analysis, and visualisation; I.M.Y. contributed to methodology, validation, formal analysis, and review and editing; F.S.A. contributed to software development, visualisation, data curation, and methodology; A.Y.A. contributed to modelling development, study pipeline, machine learning expertise, software development, conceptualisation, methodology, supervision, project administration, validation, formal analysis, writing of the original draft, review and editing, and correspondence for the entire clinical development, scientific development and property for the framework pipeline development. All authors read and approved the final manuscript.

## FUNDING INFORMATION

This study received no specific grant from any funding agency in the public, commercial, or not‐for‐profit sectors.

## CONFLICT OF INTEREST STATEMENT

The authors declare that they have no competing interests.

## ETHICS STATEMENT

The study protocol was approved by the institutional review board of Prince Sultan Military Medical City (PSMMC). Informed consent was waived for this registry‐based analysis due to the retrospective nature of the study and use of de‐identified data.

## CONSENT

The authors have nothing to report.

## Supporting information


**Table S1.** Complete feature definitions and specifications.

**Table S2.** Individual prediction stability assessment.

**Table S3.** Detailed sensitivity analysis results.

## Data Availability

The datasets used during the current study are available from the authors on reasonable request, subject to institutional data sharing policies and ethical approval requirements. The methodology utilised code is available in the following GitHub repository: https://github.com/drazzam/literature-informed-dkd-prediction/.
