# Supplementary material for: Literature‐informed ensemble machine learning for three‐year diabetic kidney disease risk prediction in type 2 diabetes: Development, validation, and deployment of the PSMMC NephraRisk model
Source: Diabetes Obes Metab. 2025 Dec 15;28(3):1997–2026. doi: 10.1111/dom.70385 (PMC12890761; doi:10.1111/dom.70385)
Supplement: Supplementary file 1 — Table S1. Complete feature definitions and specifications. [file DOM-28-1997-s003.docx]

**Supplementary Table 1:** Complete Feature Definitions and Specifications.

| **No.** | **Feature Name** | **Variable Type** | **Definition** | **Unit/Coding** | **Data Type** | **Missingness** | **Imputation Method** |
| --- | --- | --- | --- | --- | --- | --- | --- |
| 1 | Age | Observed | Patient age at index visit | Years (continuous) | Numeric | 0.0% | N/A |
| 2 | Gender |  | Biological sex | 0=Female, 1=Male | Binary | 0.0% | N/A |
| 3 | Ethnicity |  | Self-reported ethnicity | 0=Saudi, 1=Non-Saudi | Binary | 0.0% | N/A |
| 4 | Body Mass Index (BMI) |  | Weight (kg) / Height (m)² | kg/m² (continuous) | Numeric | 3.0% | Median + missing flag |
| 5 | Systolic Blood Pressure |  | Systolic BP measurement | mmHg (continuous) | Numeric | <1% | Median + missing flag |
| 6 | Diastolic Blood Pressure |  | Diastolic BP measurement | mmHg (continuous) | Numeric | <1% | Median + missing flag |
| 7 | eGFR |  | Estimated glomerular filtration rate (CKD-EPI 2021) | mL/min/1.73m² | Numeric | 0.0% | N/A |
| 8 | Albumin-Creatinine Ratio (ACR) |  | Urine albumin-to-creatinine ratio | mg/g (continuous) | Numeric | 9.4% | Median + missing flag |
| 9 | Hemoglobin A1c (HbA1c) |  | Glycemic control biomarker | % (continuous) | Numeric | 38.0% | Median + missing flag |
| 10 | Serum Phosphorus |  | Serum phosphorus level | mg/dL (continuous) | Numeric | <5% | Median + missing flag |
| 11 | FGF-23 |  | Fibroblast growth factor 23 | pg/mL (continuous) | Numeric | 15% | Median + missing flag |
| 12 | Diabetes Duration |  | Years since diabetes diagnosis | Years (continuous) | Numeric | <1% | Median + missing flag |
| 13 | Current Smoking Status |  | Active smoking at baseline | 0=No, 1=Yes, 2=Former | Categorical | <2% | Median + missing flag |
| 14 | Medication Compliance Score |  | Adherence to prescribed regimen | 0-10 scale (0=non-adherent) | Ordinal | 5% | Median + missing flag |
| 15 | CKD Stage at Baseline |  | Baseline chronic kidney disease stage | 0=No CKD, 1=Stage 1, 2=Stage 2, 3=Stage 3a, 4=Stage 3b, 5=Stage 4 | Ordinal | 0.0% | Calculated from eGFR |
| 16 | Hypertension Diagnosis |  | Clinical diagnosis of hypertension | 0=No, 1=Yes | Binary | 0.0% | N/A |
| 17 | Cardiovascular Disease History |  | Prior CVD event (MI, stroke, HF) | 0=No, 1=Yes | Binary | <1% | Mode + missing flag |
| 18 | Family History of CKD | Lit-informed imputed | First-degree relative with ESRD/CKD | 0=No, 1=Yes, 9=Unknown | Categorical | 100% | Bayesian MICE (21.8% prevalence prior from REGARDS) |
| 19 | Chronic NSAID Use |  | Regular NSAID use ≥90 days/year | 0=No, 1=Yes | Binary | 100% | Bayesian MICE (30% prevalence, HR 1.32 from NHIRD) |
| 20 | Socioeconomic Deprivation (IMD) |  | Index of Multiple Deprivation quintile | 1=Least deprived to 5=Most deprived | Ordinal | 100% | Bayesian MICE (uniform quintiles from UK CPRD) |
| 21 | Diabetic Retinopathy Severity |  | DR severity grade | 0=None, 1=Mild, 2=Moderate, 3=Severe, 4=Proliferative | Ordinal | 100% | Bayesian MICE (HR ladder [2.9, 5.8, 10.2, 16.6] from meta-analysis) |
| 22 | SGLT2 Inhibitor Use |  | Prescribed SGLT2i therapy | 0=No, 1=Yes | Binary | 100% | Bayesian MICE (HR 0.61 protective from CREDENCE/DAPA-CKD) |
| 23 | ACE Inhibitor/ARB Therapy |  | Prescribed RAAS blockade | 0=No, 1=Yes | Binary | 100% | Bayesian MICE (HR 0.77 protective from RENAAL/IDNT) |
| 24 | Statin Therapy |  | Prescribed lipid-lowering statin | 0=No, 1=Yes | Binary | 100% | Bayesian MICE (HR 0.88 protective from Chinese cohort) |
| 25 | GLP-1 Receptor Agonist Therapy |  | Prescribed GLP-1 RA | 0=No, 1=Yes | Binary | 100% | Bayesian MICE (HR 0.79 protective from FLOW trial) |

***Abbreviations:*** *ACE, angiotensin-converting enzyme; ARB, angiotensin receptor blocker; BP, blood pressure; CKD, chronic kidney disease; CKD-EPI, Chronic Kidney Disease Epidemiology Collaboration; CPRD, Clinical Practice Research Datalink; CVD, cardiovascular disease; DR, diabetic retinopathy; eGFR, estimated glomerular filtration rate; ESRD, end-stage renal disease; FGF-23, fibroblast growth factor 23; GLP-1 RA, glucagon-like peptide-1 receptor agonist; HF, heart failure; HR, hazard ratio; IMD, Index of Multiple Deprivation; Lit-informed, literature-informed; MI, myocardial infarction; MICE, multiple imputation by chained equations; N/A, not applicable; NHIRD, National Health Insurance Research Database; NSAID, non-steroidal anti-inflammatory drug; RAAS, renin-angiotensin-aldosterone system; REGARDS, Reasons for Geographic and Racial Differences in Stroke; SGLT2i, sodium-glucose co-transporter 2 inhibitor.* ***Notes:*** *Literature-informed imputed variables (features 18-25) were 100% missing by design and imputed using Bayesian MICE with external study priors; Observed variables with <40% missingness used median imputation with missing indicator flags; All continuous variables were standardized (z-score) for model input; Binary/categorical variables were one-hot encoded for machine learning models; Imputation convergence verified with Gelman-Rubin statistics R̂ ≤1.01 for all variables.*
