# Supplementary material for: Literature‐informed ensemble machine learning for three‐year diabetic kidney disease risk prediction in type 2 diabetes: Development, validation, and deployment of the PSMMC NephraRisk model
Source: Diabetes Obes Metab. 2025 Dec 15;28(3):1997–2026. doi: 10.1111/dom.70385 (PMC12890761; doi:10.1111/dom.70385)
Supplement: Supplementary file 2 — Table S2. Individual prediction stability assessment. [file DOM-28-1997-s002.docx]

**Supplementary Table 2:** Individual Prediction Stability Assessment.

| **Subgroup** | **N** | **Median PI Width (pp)** | **IQR PI Width (pp)** | **High Stability <5pp (%)** | **Moderate Stability 5-10pp (%)** | **Low Stability >10pp (%)** | **Median Absolute Deviation** | **Coefficient of Variation** | **P-value vs. Overall** |
| --- | --- | --- | --- | --- | --- | --- | --- | --- | --- |
| **OVERALL COHORT** | 2,811 | 3.2 | 2.1-4.8 | 78.0 | 19.0 | 3.0 | 0.89 | 0.28 | Reference |
| **BY BASELINE RISK CATEGORY:** | | | | | | | | | |
| Low Risk (<5%) | 1,350 | 3.1 | 2.0-4.5 | 80.2 | 17.6 | 2.2 | 0.84 | 0.27 | 0.31 |
| Moderate Risk (5-15%) | 900 | 3.4 | 2.3-5.1 | 76.1 | 20.4 | 3.5 | 0.93 | 0.29 | 0.31 |
| High Risk (15-30%) | 450 | 3.0 | 1.9-4.6 | 78.9 | 18.7 | 2.4 | 0.87 | 0.27 | 0.31 |
| Very High Risk (>30%) | 111 | 3.3 | 2.2-5.0 | 75.7 | 21.6 | 2.7 | 0.96 | 0.30 | 0.28 |
| **BY GENDER:** | | | | | | | | | |
| Male | 1,215 | 3.1 | 2.0-4.7 | 79.3 | 18.2 | 2.5 | 0.86 | 0.27 | 0.42 |
| Female | 1,596 | 3.3 | 2.2-4.9 | 76.9 | 19.7 | 3.4 | 0.91 | 0.29 | 0.42 |
| **BY AGE GROUP:** | | | | | | | | | |
| <50 years | 478 | 3.0 | 1.9-4.5 | 80.8 | 17.4 | 1.8 | 0.83 | 0.26 | 0.18 |
| 50-64 years | 1,457 | 3.2 | 2.1-4.8 | 77.8 | 19.3 | 2.9 | 0.89 | 0.28 | 0.18 |
| ≥65 years | 876 | 3.4 | 2.3-5.0 | 76.5 | 20.1 | 3.4 | 0.92 | 0.29 | 0.18 |
| **BY ETHNICITY:** | | | | | | | | | |
| Saudi | 2,800 | 3.2 | 2.1-4.8 | 78.1 | 18.9 | 3.0 | 0.89 | 0.28 | Reference |
| Non-Saudi | 11 | 3.0 | 1.8-4.3 | 81.8 | 18.2 | 0.0 | 0.79 | 0.25 | 0.72 |
| **BY CKD STAGE AT BASELINE:** | | | | | | | | | |
| No CKD (eGFR ≥90) | 1,350 | 3.0 | 1.9-4.4 | 81.0 | 17.1 | 1.9 | 0.82 | 0.26 | 0.09 |
| Stage 1-2 (eGFR 60-89) | 845 | 3.2 | 2.1-4.7 | 77.6 | 19.6 | 2.8 | 0.88 | 0.28 | 0.09 |
| Stage 3a (eGFR 45-59) | 405 | 3.4 | 2.3-5.0 | 75.3 | 21.2 | 3.5 | 0.94 | 0.30 | 0.09 |
| Stage 3b (eGFR 30-44) | 168 | 3.5 | 2.4-5.2 | 74.4 | 21.4 | 4.2 | 0.97 | 0.31 | 0.09 |
| Stage 4 (eGFR 15-29) | 43 | 3.6 | 2.5-5.4 | 72.1 | 23.3 | 4.6 | 1.01 | 0.32 | 0.09 |
| **BY HbA1c CONTROL:** | | | | | | | | | |
| Well-controlled (<7%) | 623 | 3.0 | 1.9-4.4 | 81.2 | 16.9 | 1.9 | 0.81 | 0.26 | 0.04 |
| Moderate control (7-9%) | 1,567 | 3.2 | 2.1-4.8 | 78.1 | 19.1 | 2.8 | 0.89 | 0.28 | 0.04 |
| Poor control (≥9%) | 621 | 3.5 | 2.4-5.2 | 74.6 | 21.4 | 4.0 | 0.96 | 0.30 | 0.04 |
| **BY DIABETES DURATION:** | | | | | | | | | |
| <5 years | 702 | 2.9 | 1.8-4.2 | 82.1 | 16.4 | 1.5 | 0.78 | 0.25 | 0.02 |
| 5-10 years | 1,124 | 3.2 | 2.1-4.8 | 77.9 | 19.3 | 2.8 | 0.89 | 0.28 | 0.02 |
| 10-15 years | 702 | 3.4 | 2.3-5.0 | 76.2 | 20.4 | 3.4 | 0.93 | 0.29 | 0.02 |
| ≥15 years | 283 | 3.7 | 2.5-5.5 | 72.8 | 22.6 | 4.6 | 1.03 | 0.32 | 0.02 |
| **BY PROTECTIVE MEDICATION USE:** | | | | | | | | | |
| On SGLT2i | 845 | 3.1 | 2.0-4.6 | 79.4 | 18.3 | 2.3 | 0.85 | 0.27 | 0.15 |
| Not on SGLT2i | 1,966 | 3.3 | 2.2-4.9 | 77.3 | 19.4 | 3.3 | 0.91 | 0.29 | 0.15 |
| On ACE/ARB | 1,890 | 3.2 | 2.1-4.7 | 78.3 | 18.8 | 2.9 | 0.88 | 0.28 | 0.67 |
| Not on ACE/ARB | 921 | 3.3 | 2.2-5.0 | 77.4 | 19.5 | 3.1 | 0.91 | 0.29 | 0.67 |
| **EXTREME SUBGROUPS (Intersectionality):** | | | | | | | | | |
| Male, <65y, No CKD, HbA1c <7% | 287 | 2.8 | 1.7-4.0 | 84.3 | 14.6 | 1.1 | 0.75 | 0.24 | <0.001 |
| Female, ≥65y, CKD 3b-4, HbA1c ≥9% | 92 | 4.1 | 2.8-6.2 | 67.4 | 26.1 | 6.5 | 1.15 | 0.35 | <0.001 |
| High-risk with protective Rx | 198 | 3.2 | 2.1-4.8 | 78.8 | 18.7 | 2.5 | 0.88 | 0.28 | 0.94 |
| High-risk without protective Rx | 252 | 3.7 | 2.5-5.5 | 72.2 | 23.4 | 4.4 | 1.02 | 0.32 | 0.006 |

***Abbreviations:*** *ACE, angiotensin-converting enzyme; ARB, angiotensin receptor blocker; CKD, chronic kidney disease; eGFR, estimated glomerular filtration rate; HbA1c, hemoglobin A1c; IQR, interquartile range; N, number of patients; PI, prediction interval; pp, percentage points; Rx, therapy; SGLT2i, sodium-glucose co-transporter 2 inhibitor.* ***Statistical Methods:*** *Individual prediction stability assessed via 1,000 bootstrap resamples per patient following Riley & Collins (2023) methodology. Prediction interval (PI) width calculated as the range between 2.5th and 97.5th percentiles of bootstrapped predictions. Coefficient of variation = standard deviation / mean of bootstrapped predictions. Median absolute deviation = median of |prediction - median prediction| across bootstrap samples. Stability categories: High (<5 percentage points), Moderate (5-10 percentage points), Low (>10 percentage points). P-values from Kruskal-Wallis test comparing PI width distributions vs. overall cohort, with Bonferroni correction for multiple comparisons (significance threshold p<0.05).*
