# Supplementary material for: Literature‐informed ensemble machine learning for three‐year diabetic kidney disease risk prediction in type 2 diabetes: Development, validation, and deployment of the PSMMC NephraRisk model
Source: Diabetes Obes Metab. 2025 Dec 15;28(3):1997–2026. doi: 10.1111/dom.70385 (PMC12890761; doi:10.1111/dom.70385)
Supplement: Supplementary file 3 — Table S3. Detailed sensitivity analysis results. [file DOM-28-1997-s001.docx]

**Supplementary Table 3:** Detailed Sensitivity Analysis Results.

| **Analysis Category** | **Scenario Description** | **N** | **AUROC (95% CI)** | **Δ-AUROC vs. Base** | **Calibration Slope** | **Brier Score** | **P-value** | **Clinical Interpretation** |
| --- | --- | --- | --- | --- | --- | --- | --- | --- |
| **BASE MODEL** | Final clinical model (reference) | 2,811 | 0.852 (0.847-0.857) | Reference | 0.98 | 0.085 | — | Reference performance |
| **LITERATURE PRIOR STRENGTH:** | | | | | | | | |
| Conservative priors | All priors at 50% of literature effect sizes | 2,811 | 0.847 (0.842-0.852) | -0.005 | 0.96 | 0.087 | 0.06 | Minimal degradation with weakened priors |
| Optimistic priors | All priors at 150% of literature effect sizes | 2,811 | 0.857 (0.852-0.862) | +0.005 | 1.00 | 0.084 | 0.04 | Slight improvement with strengthened priors |
| Flat non-informative priors | Uniform priors (no literature information) | 2,811 | 0.844 (0.839-0.849) | -0.008 | 0.94 | 0.088 | <0.001 | Literature priors provide little but significant benefit |
| Asian-specific priors only | Using only Asian population effect sizes (7 studies) | 2,811 | 0.850 (0.845-0.855) | -0.002 | 0.97 | 0.086 | 0.38 | Ethnic-specific priors show minimal difference |
| White-specific priors only | Using only White population effect sizes (16 studies) | 2,811 | 0.849 (0.844-0.854) | -0.003 | 0.97 | 0.086 | 0.24 | Confirms significance across ethnic sources |
| **MISSING DATA HANDLING:** | | | | | | | | |
| Complete case analysis | Only patients with all observed variables | 1,740 | 0.849 (0.843-0.855) | -0.003 | 0.97 | 0.083 | 0.34 | Imputation approach validated |
| HbA1c: Complete cases only | Only patients with observed HbA1c (n=1,740) | 1,740 | 0.849 (0.843-0.855) | -0.003 | 0.97 | 0.083 | 0.34 | HbA1c imputation method appropriate |
| HbA1c: MICE imputation | Alternative MICE for HbA1c vs. median | 2,811 | 0.851 (0.846-0.856) | -0.001 | 0.98 | 0.085 | 0.68 | Imputation method choice minimal impact |
| HbA1c: Mean imputation | Mean imputation without missing flag | 2,811 | 0.848 (0.843-0.853) | -0.004 | 0.96 | 0.086 | 0.12 | Missing indicator adds information |
| High missingness exclusion | Exclude all variables >30% missing | 2,811 | 0.846 (0.841-0.851) | -0.006 | 0.96 | 0.087 | 0.02 | High-missingness variables contribute |
| Waist circumference observed only | Complete cases for waist circumference (n=1,358) | 1,358 | 0.854 (0.847-0.861) | +0.002 | 0.99 | 0.082 | 0.52 | Imputed waist circumference appropriate |
| ACR: High missingness subset | Patients with ACR missing (n=264) | 264 | 0.834 (0.815-0.853) | -0.018 | 0.93 | 0.091 | 0.08 | Acceptable performance in high-missing subset |
| All observed variables only | Exclude all literature-informed variables | 2,811 | 0.842 (0.837-0.847) | -0.010 | 0.96 | 0.088 | <0.001 | Literature-informed variables add significant value |
| **VARIABLE EXCLUSION ANALYSES:** | | | | | | | | |
| Exclude socioeconomic (IMD) | Remove IMD quintiles from model | 2,811 | 0.849 (0.844-0.854) | -0.003 | 0.98 | 0.085 | 0.26 | IMD contributes minimally (1.4% clinical gain) |
| Exclude waist circumference | Remove waist circumference from model | 2,811 | 0.850 (0.845-0.855) | -0.002 | 0.98 | 0.085 | 0.42 | Waist circumference minimal unique contribution |
| Exclude family history | Remove family history of CKD | 2,811 | 0.848 (0.843-0.853) | -0.004 | 0.97 | 0.086 | 0.14 | Family history adds modest value |
| Exclude NSAID exposure | Remove chronic NSAID use variable | 2,811 | 0.850 (0.845-0.855) | -0.002 | 0.98 | 0.085 | 0.48 | NSAID minimal independent effect |
| Exclude diabetic retinopathy | Remove DR severity (3rd most important) | 2,811 | 0.837 (0.832-0.842) | -0.015 | 0.95 | 0.089 | <0.001 | DR severity critical predictor |
| Exclude all protective medications | Remove SGLT2i, ACE/ARB, statins, GLP-1 RA | 2,811 | 0.845 (0.840-0.850) | -0.007 | 0.96 | 0.087 | 0.008 | Protective medications collectively important |
| eGFR and ACR only | Minimal model with top 2 predictors only | 2,811 | 0.814 (0.809-0.819) | -0.038 | 0.92 | 0.095 | <0.001 | Additional features provide substantial benefit |
| Clinical variables only | Exclude all biomarkers except eGFR/ACR | 2,811 | 0.826 (0.821-0.831) | -0.026 | 0.94 | 0.091 | <0.001 | Laboratory biomarkers essential |
| **COMPETING RISK MODELS:** | | | | | | | | |
| Fine-Gray subdistribution | Account for competing risk of death | 2,811 | 0.853 (0.848-0.858) | +0.001 | 0.98 | 0.085 | 0.71 | Competing risk handling minimal impact |
| Cause-specific hazard | Death as censoring event | 2,811 | 0.852 (0.847-0.857) | 0.000 | 0.98 | 0.085 | 0.95 | Consistent with primary analysis |
| Exclude deaths from analysis | Remove patients who died before 36mo | 2,698 | 0.851 (0.846-0.856) | -0.001 | 0.98 | 0.084 | 0.72 | Death exclusion does not alter findings |
| Transplant as competing event | Kidney transplant as competing risk | 2,811 | 0.852 (0.847-0.857) | 0.000 | 0.98 | 0.085 | 0.88 | Transplant events rare, minimal impact |
| **TEMPORAL VALIDATION VARIATIONS:** | | | | | | | | |
| Alternative temporal split | Training ≤Jun 2020, Validation Jul-Dec 2020, Test 2021 | 2,811 | 0.848 (0.843-0.853) | -0.004 | 0.97 | 0.086 | 0.18 | Temporal split choice significant |
| No validation set | Direct train-test split (80-20) | 2,811 | 0.856 (0.851-0.861) | +0.004 | 1.01 | 0.083 | 0.11 | Validation set prevents overfitting appropriately |
| Random split | Non-temporal random 70-15-15 split | 2,811 | 0.867 (0.862-0.872) | +0.015 | 1.04 | 0.082 | <0.001 | Temporal split appropriately conservative |
| Test on most recent 6mo | Test set = Jan-Jun 2022 only | 1,405 | 0.854 (0.847-0.861) | +0.002 | 0.99 | 0.084 | 0.58 | Consistent performance on recent data |
| Test on earliest 6mo | Test set = Jul-Dec 2021 only | 1,406 | 0.850 (0.843-0.857) | -0.002 | 0.97 | 0.086 | 0.54 | Stable across temporal test periods |
| **OUTCOME DEFINITION VARIATIONS:** | | | | | | | | |
| Stricter DKD definition | eGFR <60 with ≥40% decline (vs. ≥25%) | 2,811 | 0.869 (0.864-0.874) | +0.017 | 1.01 | 0.078 | <0.001 | Better discrimination for severe outcomes |
| More lenient DKD definition | eGFR <60 with ≥15% decline | 2,811 | 0.837 (0.832-0.842) | -0.015 | 0.95 | 0.091 | <0.001 | Reduced discrimination for milder outcomes |
| ACR progression only | Outcome = ACR increase ≥30% only | 2,811 | 0.823 (0.818-0.828) | -0.029 | 0.93 | 0.093 | <0.001 | eGFR decline better predicted than albuminuria |
| eGFR decline only | Outcome = eGFR decline only, not ACR | 2,811 | 0.841 (0.836-0.846) | -0.011 | 0.96 | 0.088 | <0.001 | Composite outcome optimal |
| ESRD only | Outcome = progression to dialysis/ESRD | 2,811 | 0.891 (0.878-0.904) | +0.039 | 1.03 | 0.045 | <0.001 | Excellent discrimination for ESRD (rare event) |
| Any CKD progression | Outcome = any worsening of CKD stage | 2,811 | 0.828 (0.823-0.833) | -0.024 | 0.94 | 0.094 | <0.001 | Broad definition reduces specificity |
| **SUBGROUP ANALYSES:** | | | | | | | | |
| Age <65 years only | Younger subset | 1,935 | 0.847 (0.841-0.853) | -0.005 | 0.96 | 0.087 | 0.08 | Consistent performance in younger patients |
| Age ≥65 years only | Older subset | 876 | 0.857 (0.849-0.865) | +0.005 | 1.00 | 0.083 | 0.08 | Slightly better in older patients |
| Male only | Male subset | 1,215 | 0.854 (0.847-0.861) | +0.002 | 0.99 | 0.084 | 0.52 | Sex-specific performance excellent |
| Female only | Female subset | 1,596 | 0.850 (0.843-0.857) | -0.002 | 0.97 | 0.086 | 0.52 | Minimal gender difference |
| No baseline CKD | eGFR ≥90 at baseline | 1,350 | 0.832 (0.825-0.839) | -0.020 | 0.94 | 0.089 | <0.001 | Lower discrimination in healthy kidneys |
| Baseline CKD Stage 3+ | eGFR <60 at baseline | 616 | 0.871 (0.862-0.880) | +0.019 | 1.02 | 0.079 | <0.001 | Better discrimination in established CKD |
| HbA1c <7% (well-controlled) | Good glycemic control | 623 | 0.838 (0.829-0.847) | -0.014 | 0.95 | 0.088 | 0.002 | Reduced discrimination in well-controlled |
| HbA1c ≥9% (poor control) | Poor glycemic control | 621 | 0.863 (0.854-0.872) | +0.011 | 1.01 | 0.082 | 0.002 | Better discrimination in poor control |
| On SGLT2i at baseline | Protected subset | 845 | 0.848 (0.839-0.857) | -0.004 | 0.97 | 0.086 | 0.28 | Consistent in treated patients |
| No protective medications | Unprotected subset | 432 | 0.857 (0.846-0.868) | +0.005 | 1.00 | 0.083 | 0.28 | Good discrimination identifies need for Rx |
| **MODEL ARCHITECTURE VARIATIONS:** | | | | | | | | |
| Logistic regression (no ML) | Simple logistic with all features | 2,811 | 0.804 (0.799-0.809) | -0.048 | 0.98 | 0.094 | <0.001 | Machine learning provides substantial benefit |
| LightGBM only (no ensemble) | Single LightGBM model | 2,811 | 0.862 (0.857-0.867) | +0.010 | 1.05 | 0.082 | <0.001 | Ensemble improves calibration |
| CoxBoost only (no ensemble) | Single survival model | 2,811 | 0.849 (0.844-0.854) | -0.003 | N/A | 0.084 | 0.26 | Ensemble optimal |
| XGBoost alternative | XGBoost instead of LightGBM | 2,811 | 0.858 (0.853-0.863) | +0.006 | 1.03 | 0.083 | 0.02 | LightGBM+CoxBoost ensemble superior |
| Random Forest alternative | Random Forest classifier | 2,811 | 0.846 (0.841-0.851) | -0.006 | 0.96 | 0.087 | 0.03 | Boosting methods outperform bagging |
| Neural network alternative | 2-layer feed-forward network | 2,811 | 0.853 (0.848-0.858) | +0.001 | 0.99 | 0.085 | 0.76 | Comparable but not superior to ensemble |
| Simple averaging ensemble | Mean of LightGBM + CoxBoost (no meta-learner) | 2,811 | 0.860 (0.855-0.865) | +0.008 | 1.02 | 0.083 | 0.007 | Meta-learner provides calibration benefit |
| Weighted ensemble (AUC weights) | Weights based on individual model AUROCs | 2,811 | 0.863 (0.858-0.868) | +0.011 | 1.04 | 0.082 | <0.001 | Learned weights via meta-learner optimal |
| **CALIBRATION VARIATIONS:** | | | | | | | | |
| No calibration | Raw ensemble predictions | 2,811 | 0.866 (0.861-0.871) | +0.014 | 1.06 | 0.087 | <0.001 | Calibration essential for clinical use |
| Platt scaling | Logistic calibration | 2,811 | 0.852 (0.847-0.857) | 0.000 | 0.99 | 0.085 | 0.88 | Similar to isotonic calibration |
| Beta calibration | Beta distribution calibration | 2,811 | 0.852 (0.847-0.857) | 0.000 | 0.98 | 0.085 | 0.94 | Isotonic calibration optimal |
| Validation set calibration only | No test set recalibration | 2,811 | 0.852 (0.847-0.857) | 0.000 | 0.98 | 0.085 | 0.97 | Single calibration step sufficient |

***Abbreviations:*** *ACE, angiotensin-converting enzyme; ACR, albumin-creatinine ratio; ARB, angiotensin receptor blocker; AUROC, area under receiver operating characteristic curve; CI, confidence interval; CKD, chronic kidney disease; DKD, diabetic kidney disease; DR, diabetic retinopathy; eGFR, estimated glomerular filtration rate; ESRD, end-stage renal disease; GLP-1 RA, glucagon-like peptide-1 receptor agonist; HbA1c, hemoglobin A1c; IMD, Index of Multiple Deprivation; MICE, multiple imputation by chained equations; ML, machine learning; N, sample size; N/A, not applicable; NSAID, non-steroidal anti-inflammatory drug; Rx, therapy; SGLT2i, sodium-glucose co-transporter 2 inhibitor; vs., versus.* ***Statistical Methods:*** *P-values from DeLong test comparing AUROC to base model. Δ-AUROC = difference from base model (0.852). Significance threshold p<0.05 with Bonferroni correction for multiple comparisons within each analysis category. Bootstrap 95% confidence intervals calculated using 1,000 bias-corrected accelerated resamples.*
